# Pentoxifylline Inhibits Pulmonary Fibrosis by Regulating Cellular Senescence in Mice

**DOI:** 10.3389/fphar.2022.848263

**Published:** 2022-05-19

**Authors:** Yifan Lin, Zhihao Xu, Beibei Zhou, Keer Ma, Mengyi Jiang

**Affiliations:** Department of Respiratory and Critical Care Medicine, The Fourth Affiliated Hospital, School of Medicine, Zhejiang University, Yiwu, China

**Keywords:** pentoxifylline, idiopathic pulmonary fibrosis, pulmonary fibrosis, interstitial lung disease, cellular senescence, senolytics, pharmacological treatment, antifibrotic

## Abstract

Idiopathic pulmonary fibrosis (IPF) is a chronic progressive disease, and its occurrence and development are mediated by cellular senescence. Drugs targeting senescent cells seem like a promising and efficacious strategy for IPF treatment. Previous studies have illustrated that pentoxifylline (PTX) may play a certain role in inhibiting pulmonary fibrosis and combating cellular senescence. In this study, we demonstrated that PTX administration inhibits pulmonary fibrosis development and cellular senescence in the bleomycin (BLM)-induced IPF mice model. Moreover, the expression levels of fibrosis-related genes and senescence-related genes in mice lung tissue and primary pulmonary fibroblasts illustrated lung fibroblasts’ vital role in these two processes. And the curative effect of PTX was completed mainly by acting on lung fibroblasts. Besides, during the whole treatment, delayed initiation or advanced halt of PTX administration would influence its effectiveness in reducing fibrotic and senescent traits in various degrees, and the latter influenced more. We further determined that a long period of PTX administration could bring noticeable benefits to mice in recovering BLM-induced lung fibrosis and suppressing age-associated cellular senescence. Moreover, it was still effective when PTX administration was used to treat senescent human fibroblasts. Thus, our findings manifested that PTX therapy is an efficient remedy for pulmonary fibrosis by suppressing cellular senescence.

## Introduction

Idiopathic pulmonary fibrosis (IPF) is a chronic interstitial lung disease. The severe deposition of extracellular matrix in the lung interstitium and the gradual deterioration of lung function resultantly are dominant characteristics of IPF disease (Raghu et al., 2011). The etiology of IPF is still unclear, but researchers have noticed that there are multiple risk factors for IPF, including genetic inheritance, aging, gender, smoking history, environmental exposure, and gastroesophageal reflux (Iwai et al., 1994; Baumgartner et al., 2000; Raghu et al., 2011). Moreover, among all these risk factors, aging is an important one.

On the one hand, generous epidemiological information has confirmed that IPF is an aging-related disease (Choi et al., 2018). The prevalence of IPF in the population increases with age, and the interstitial changes of these IPF patients in chest imaging also increase with physiological age (Raghu et al., 2006; Fell et al., 2010; Hutchinson et al., 2014). On the other hand, primary pulmonary fibroblasts isolated from the lung tissue of IPF patients perform more senescent features in morphology and physiological function than primary fibroblasts isolated from the lung tissue of age-matched control. This change is consistent with the view that aging is based on the accumulation of senescent cells (Álvarez et al., 2017; Gorgoulis et al., 2019). According to our previous perspectives, fibroblasts are direct executors of the fibrosis process, and senescent fibroblasts mediate the occurrence and development of IPF. Fibroblasts in IPF show significant characteristics of cellular senescence such as abnormal activation, metabolic transformation, mitochondria dysfunction, apoptosis resistance, autophagy defect, and increased senescence-associated secretory phenotype (SASP) (Lin and Xu, 2020).

With the in-depth study of cellular senescence in IPF, researchers gradually realized that the clearance of senescent cells could be regarded as a new strategy for treating IPF therapy. Furthermore, researchers have been searching for drugs that can execute anti-senescence works from multiple natural and manufactured compounds. The combined use of dasatinib and quercetin as a senolytic cocktail could selectively remove senescent fibroblasts from a bleomycin-induced IPF mouse model, thus delaying the progress of pulmonary fibrosis in mice with excellent safety and efficiency (Baker et al., 2011; Schafer et al., 2017). In addition, this senolytic cocktail significantly improved 14 IPF clinical patients’ lung function and physical condition, accompanied by an excellent tolerance (Justice et al., 2019). Clinical drugs, such as pirfenidone, nintedanib, and metformin, as well as active extracts from natural plants, such as fisetin and phloretin, were both essential sources for discovering new anti-senescence drugs because of their pleiotropy and safety (Rangarajan et al., 2016; Cho et al., 2017; Rangarajan et al., 2018; Yousefzadeh et al., 2018; Ballester et al., 2020; Lin and Xu, 2020). And in this study, our attention turns to an “old drug,” pentoxifylline.

Pentoxifylline (PTX) is a dimethyl xanthine derivative, an alkaloid obtained by introducing the hexone group into the theobromine. PTX is often used in clinical therapy as a nonspecific peripheral vasodilator because of its nonselective inhibition of phosphodiesterase. Besides, PTX has an outstanding anti-fibrosis performance in animal experiments and clinical trials. It is worth noting that in those strictly designed clinical trials, PTX was very effective to alleviate radiotherapy-related fibrosis and even osteonecrosis when treating a variety of cancer patients, including non-small cell lung cancer, pelvic cancer, head and neck squamous cell carcinoma, breast cancer, and so on (Kwon et al., 2000; Aygenc et al., 2004; Gothard et al., 2005; Haddad et al., 2005; Delanian et al., 2011; Jacobson et al., 2013; Cook et al., 2016). PTX was helpful to treat abnormal superficial fibrosis such as skin hypertrophic scar and oral submucosal fibrosis, and it also contributed to controling the development of organ fibrosis such as liver fibrosis, systemic sclerosis, and cystic fibrosis (Futran et al., 1997; de Souza et al., 2009; Alam et al., 2017; Sadaksharam and Mahalingam, 2017; Kholakiya et al., 2020; Tan et al., 2020; Kedarisetty et al., 2021; Serag-Eldin et al., 2021). For the specific mechanisms behind this anti-fibrosis effect, some researchers have illustrated that PTX treatment was capable of inhibiting the proliferation and extracellular matrix production of fibroblasts (Kaya et al., 2014; Lee et al., 2017). PTX effectively reduced the expression of plasminogen activator inhibitor-1 (PAI-1) and fibronectin (Fn) in irradiated rat lung tissue and lung epithelial cells by restoring the phosphorylation of protein kinase A (PKA) (Ward et al., 1992; Lee et al., 2017). And what interests us was that PAI-1 and PKA were also associated with the significant signaling pathways about how senescent fibroblasts affect IPF, which indicated that PTX might regulate the occurrence and development of pulmonary fibrosis by mediating cellular senescence (Lin and Xu, 2020).

Concerning the inhibitory effect of PTX on cellular senescence, we have sufficient evidence to prove its feasibility. Studies have shown that the vasorelaxant effects of PTX depended on animals’ age and fundamental vasomotor tension. That is to say, the response of animals to PTX treatment was more significant when they were older (Kaapa et al., 1991; Raj et al., 1992). Erythrocytes isolated from young and aged rats were also different in their sensitivity to PTX treatment (Seidler and Swislocki, 1992). More specifically, PTX improved natural aging rats’ learning and memory ability and significantly reversed the rats’ age-dependent spatial memory defect (de Toledo-Morrell et al., 1984; Hu et al., 2007). Similar improvements also existed in clinical patients (Parnetti et al., 1986; Solerte et al., 2000; Wang et al., 2020). Moreover, PTX supplementation reduced the level of reactive oxygen species (ROS) in rat oocytes in a concentration-dependent manner, thus delaying the occurrence of post-ovulation aging (Premkumar and Chaube, 2016). Furthermore, PTX reduced cellular senescence induced by chemotherapeutic drugs such as Cisplatin and Adriamycin in tumor cells, increasing cell apoptosis and reducing cell viability after chemotherapy (Bravo-Cuellar et al., 2010; Hernandez-Flores et al., 2011). Therefore, we have reasons to expect the practicability of PTX in suppressing pulmonary fibrosis by reducing cellular senescence.

We first used bleomycin (BLM) to induce pulmonary fibrosis in mice as an IPF model to verify our hypothesis. The construction of this BLM model also led to cellular senescence in mice. PTX administration with different onset times and duration was used to verify the anti-fibrosis and anti-senescence effects of PTX treatment, emphasizing on the expressions of fibrosis-related and senescence-related genes in lung tissue and pulmonary fibroblasts. The results showed that mice with pulmonary fibrosis benefit from timely and sustained PTX administration. PTX suppressed the formation of pulmonary fibrosis and retarded cellular senescence in the progression of pulmonary fibrosis and age growth. When PTX was used in treating senescent human lung fibroblast HFL1 cells, the anti-senescence effects of PTX still existed, so did the anti-fibrotic effects. This result suggested that the therapeutic effect of PTX might also apply to humans. Our findings provided substantial evidence for PTX as a great attempt to treat pulmonary fibrosis and concurrent cellular senescence.

## Materials and Methods

### Mouse Model

**FIGURE 1:**
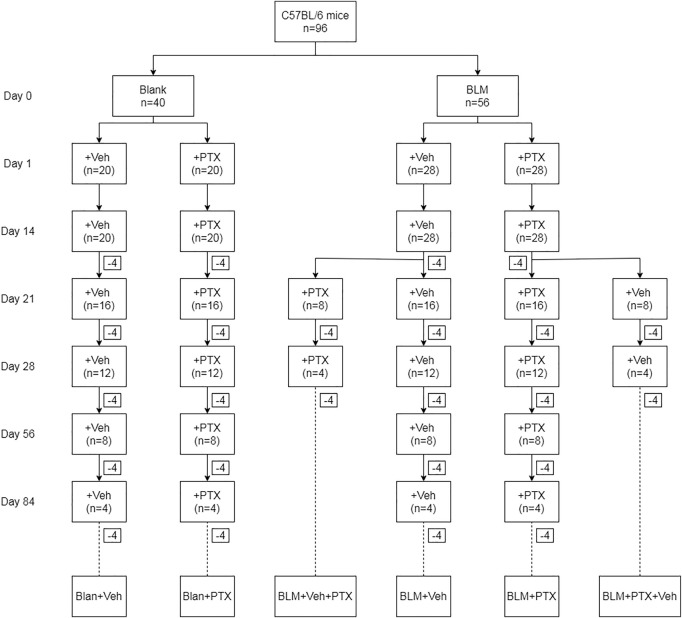
The animal grouping and interventions are briefly indicated. “Blank” represents for the intratracheal instillation of sterile normal saline on day 0, and “BLM” represents for the intratracheal instillation of bleomycin on day 0. “Veh” represents for the blank treatment in the experiments, and “PTX” represents for the PTX treatment in the experiments.

On day 0, anesthetized C57BL/6 male mice (*n* = 96; 6–8 weeks, 20–25 g) were randomly divided according to the grouping shown in Figure 1. Mice in the BLM group were given an intratracheal injection with bleomycin (BLM, 2 mg/kg; CAS: 9041-93-4, Aladdin), while mice in the Blank group received the same amount of sterile normal saline. Intragastric administration of pentoxifylline (PTX, 1.5 mg per mouse; CAS: 6493-05-6, Aladdin) or vehicle (Veh) commenced on the next day of BLM instillation (Day 1), once a day. According to the treatment received by mice, they were recorded as Blank + Veh group, BLM + Veh group, BLM + PTX group, and Blank + PTX group, respectively. Moreover, to study the influence of PTX administration timing on its efficacy, on day 14, mice in the BLM + Veh group were randomly divided into two groups again. Mice of one group continued the Veh treatment from day 15 to day 28, while mice of the other group changed their treatment into PTX from day 15 to day 28, and the mice that changed their treatment were recorded as BLM + Veh + PTX group. So was in the BLM + PTX group, and the mice changing their treatment into the vehicle were recorded as BLM + PTX + Veh group. All mice were weighed on day 0, day 7, day 14, day 21, and day 28. Moreover, on day 14, day 21, and day 28, 4 mice of each group were sacrificed. After day 28, the remaining mice in each group continued their initial treatment and were then sacrificed on day 56 and day 84.

### Cell Culture and Treatment

Primary mice lung fibroblasts were harvested from lung tissues obtained from sacrificed mice in each group on day 14, day 21, and day 28 (Roman et al., 2005; Seluanov et al., 2010). The left upper lobe of each sacrificed mouse was taken out in the super clean bench. Cut off the connective tissue at the hilum, and wash the blood on the surface away with phosphate-buffered saline (PBS) until it turns clean. Then the lung tissue was entirely cut into scraps together with 100 μl fetal bovine serum (FBS; 10270-106, Thermo Fisher Scientific) by sterile ophthalmic scissors until a paste. Disposable sterile pipette tips were used to transfer lung tissue blocks and arrange them neatly at the bottom of T25 cell culture flasks, with a distance of about 3–5 mm. Invert the cell culture flasks and add 6–10 ml Dulbecco’s modified eagle medium (DMEM; 11965-118; Thermo Fisher Scientific) with high glucose, containing 10–12% FBS and 1% penicillin-streptomycin (15140-122; Thermo Fisher Scientific). The inverted cell culture flasks were carefully moved into a 37°C, 5% CO_2_ cell incubator and incubated for 2 h to make the tissue blocks slightly dry and fully attached to the bottom of the cell culture flasks. And 2 h later, carefully turn the cell culture flasks back so that the DMEM culture medium could slowly cover the bottom of the cell culture flasks without floating the tissue blocks. Change half of the DMEM culture medium every 2–3 days afterward. After 7–14 days, wedge-shaped lung fibroblasts thrived under the microscope, forming a compact monolayer. When the lung fibroblasts entirely covered the bottom of cell culture flasks, the fibroblasts were harvested by 0.25% trypsin (C0201, Beyotime), counted, and then consecutively passaged. The 3rd to 6th generations of lung fibroblasts were used for further experiments, including senescence-associated β-galactosidase (SA-β-gal) staining, RNA extraction, and protein extraction.

Human fetal lung fibroblasts HFL1 were obtained from American Type Culture Collection (ATCC). HFL1 cells were maintained in F12K (21127-022, Thermo Fisher Scientific) medium containing 10 FBS and 1% penicillin-streptomycin. Senescent HFL1 cells were introduced by etoposide (VP-16; CAS: 33419-42-0, Aladdin) treatment at a concentration of 50 mM for 48 h. HFL1 cells acquired the senescence phenotype 7 days post VP-16 treatment (Yosef et al., 2016). HLF1 and senescent HLF1 cells were plated in 12-well plates at 1.0 × 10^5^ cells per well. After overnight attachment, cells were treated with vehicle or PTX (4 mM) for 24 h and collected using trypsin. Collected cells were used for further experiments, including cell counting kit-8 assay, SA-β-gal staining, RNA extraction, and protein extraction.

### Cell Counting Kit-8 Assay

HLF1 cells and senescent HLF1 cells were plated as described above, and the vehicle or PTX treatment was added to each well at the indicated concentrations (1, 2, 3, 4, 5, 6, 7, and 8 mM) for 24 h. Twenty four hours later, cell viability was determined by WST-8 assay using Cell Counting Kit-8 (CCK-8; C0037, Beyotime), according to the manufacturer’s instructions.

### Histopathological Analysis

The right lower lobe of each sacrificed mouse was taken out and immediately embedded with optimum cutting temperature compound (OCT Compound; Sakura). After that, they were quick-frozen by a freezing microtome and then sectioned at 6 μm thickness. The frozen sections of mice lung tissue were performed hematoxylin-eosin staining (HE staining; C0105S, Beyotime) and Masson’s trichrome staining (Masson staining; G1340, Solarbio) to observe the pathologic morphological changes in the lung tissue, according to the manufacturer’s instructions. The fibrosis levels were evaluated through the Ashcroft scoring system (Ashcroft et al., 1988). In addition, software ImageJ was used to analyze the Masson staining results and evaluated the collagen volume fraction (CVF), the ratio of the blue area after Masson staining to the total tissue area.

### Hydroxyproline Content

The hydroxyproline (HYP) content in the mice lung tissue and the cultured supernatants of HFL1 cells were detected by the HYP content detection kit (BC0255, Solarbio), according to the manufacturer’s instructions. Briefly, the mice lung tissue and cultured supernatants of HFL1cells were fully hydrolyzed by 6 mol/L hydrochloric acid at 100°C for 6–8 h. Then the hydrolysis product was oxidized by chloramine T and further reacted with p-dimethylaminobenzaldehyde to produce characteristic red compounds. By measuring the absorbance value of reaction products at 560 nm wavelength by colorimetry, the HYP content of mice lung tissue and cultured supernatants of HFL1 cells were calculated. The measurement unit for the HYP content in lung tissue and cultured supernatants of HFL1 cells is μg/mg and µg/mg protein, respectively.

### Senescence-Associated β-Galactosidase Staining

The frozen sections of mice lung tissue, primary mouse lung fibroblasts, and HFL1 cells were carried out with SA-β-gal staining to evaluate the formation and accumulation of senescent cells. The SA-β-gal staining kit (C0602, Beyotime) was used to detect the high enzyme activity of β-galactosidase in senescent cells, according to the manufacturer’s instructions. Essentially, the β-galactosidase in senescent cells takes X-gal as the substrate, and the reaction is performed at pH 6.0 to produce the dark blue product, which is relatively easy to distinguish under the microscope.

### Western Blot

Mice lung tissue, primary mouse lung fibroblasts, and HFL1 cells were lysed with radio immunoprecipitation assay lysis buffer (RIPA; P0013C, Beyotime), then a BCA protein assay kit (P0012S, Beyotime) was used to determine the protein concentration of protein extract. Subsequently, the protein extract was carried out with sodium dodecyl sulfate-polyacrylamide gel electrophoresis (SDS-PAGE) and then transferred to the polyvinylidene fluoride (PVDF) membrane. The PVDF membrane was fully blocked in 5% skimmed milk afterward and used for subsequent specific immune binding reactions. First, the primary antibodies of p16 protein (sc-377412, Santa Cruz; dilution 1:1000) and β-actin protein (sc-47778, Santa Cruz; dilution 1:1000), then the horseradish peroxidase (HRP)-labeled second antibody (A0216, Beyotime) were used in these reactions. After all antibody incubation processes were completed, chemiluminescence was carried out (P0018S, Beyotime), and the obtained images were further analyzed by software ImageJ. All experiments in this part were carried out following the manufacturer’s instructions. All data were normalized to the expression level of β-actin protein.

### Real-Time Quantitative Polymerase Chain Reaction

For total RNA extraction, Trizol (R0016, Beyotime) was used for the RNA extraction from mice lung tissue, and RNAeasy™ Animal RNA Isolation Kit with Spin Column (R0027, Beyotime) was used for the RNA extraction from primary mouse lung fibroblasts and HFL1 cells. A Nanodrop machine was used to determine the concentration and purity of extracted RNA. BeyoRT™II First Strand cDNA Synthesis Kit (D7168L, Beyotime) was used to reverse messenger RNA (mRNA) to complementary DNA (cDNA). Then, real-time quantitative polymerase chain reaction (RT-qPCR) was performed using BeyoFast™ SYBR Green qPCR Mix (D7260, Beyotime). All experiments in this part were carried out following the manufacturer’s instructions, and all data were normalized to the expression level of β-actin mRNA. The primers’ sequence used in RT-qPCR is listed in Table 1.

**TABLE 1:** Primer sequence used in real-time qPCR.

Gene	Species	Direction	Sequence (5′ -> 3′)
Col1a1	Mouse	Forward	CTGGAAGAGCGGAGAGTA
		Reverse	CTGTAGGTGAAGCGACTG
Acta2	Mouse	Forward	AGAACACGGCATCATCAC
		Reverse	GCA​GTA​GTC​ACG​AAG​GAA​T
Fn1	Mouse	Forward	ATA​CCT​GCC​GAA​TGT​AGA​TG
		Reverse	GCC​TCC​ACT​ATG​ATG​TTG​TA
Tgfb1	Mouse	Forward	GCAACAACGCCATCTATG
		Reverse	CAAGGTAACGCCAGGAAT
Cdkn2a	Mouse	Forward	CCG​ATT​CAG​GTG​ATG​ATG​AT
		Reverse	CGCACGATGTCTTGATGT
Tnf	Mouse	Forward	GTGGAACTGGCAGAAGAG
		Reverse	TAGACAGAAGAGCGTGGT
Serpine1	Mouse	Forward	GCCGTGGAACAAGAATGA
		Reverse	GGT​GGT​GAA​CTC​AGT​GTA​G
Ccl2	Mouse	Forward	CAA​TGA​GTA​GGC​TGG​AGA​G
		Reverse	GAAGTGCTTGAGGTGGTT
Mmp10	Mouse	Forward	TGGAGAACACGGAGACTT
		Reverse	GAG​ACA​GAC​AAC​ACA​GGA​A
Mmp12	Mouse	Forward	ACA​ACT​CAA​CTC​TGG​CAA​T
		Reverse	CTA​CAT​CCT​CAC​GCT​TCA​T
Actin, beta	Mouse	Forward	CTCACTGTCCACCTTCCA
		Reverse	CTCCAACCAACTGCTGTC
COL1A1	Human	Forward	TGA​CTG​GAA​GAG​TGG​AGA​G
		Reverse	GTGACGCTGTAGGTGAAG
ACTA2	Human	Forward	CTT​GAG​AAG​AGT​TAC​GAG​TTG
		Reverse	GAT​GCT​GTT​GTA​GGT​GGT​T
FN1	Human	Forward	TTA​TGC​CGT​TGG​AGA​TGA​G
		Reverse	CTG​AGA​ATA​CTG​GTT​GTA​GGA
TGFB1	Human	Forward	TCA​GTC​ACC​ATA​GCA​ACA​C
		Reverse	CACCTTAGCCTCCAGAGT
CDKN2A	Human	Forward	ACCAGAGGCAGTAACCAT
		Reverse	GTAGGACCTTCGGTGACT
TNF	Human	Forward	GCAACAAGACCACCACTT
		Reverse	CTC​CAG​ATT​CCA​GAT​GTC​AG
SERPINE1	Human	Forward	CCT​TGA​GTG​CTT​GTT​AGA​GA
		Reverse	TTC​CTG​AGA​TAC​GGT​GAC​A
CCL2	Human	Forward	TAGCAGCCACCTTCATTC
		Reverse	TGT​TCA​AGT​CTT​CGG​AGT​T
MMP10	Human	Forward	GAA​GGA​GAG​GCT​GAT​ATA​ATG​A
		Reverse	CTG​TGA​ATG​AGT​TGT​AGA​GTG
MMP12	Human	Forward	CACATTCAGGAGGCACAA
		Reverse	CGT​ATG​TCA​TCA​GCA​GAG​A
ACTIN, Beta	Human	Forward	TCGTGCGTGACATTAAGG
		Reverse	AGGAAGGAAGGCTGGAAG

### Statistical Analysis

Software GraphPad Prism 8.02 was used for the statistical analysis process and diagram generation. All data presented in this research were expressed as mean ± standard deviation (SD). For multiple comparisons, the one-way analysis of variance (ANOVA) with Tukey’s post-hoc test was used. A *p* value < 0.05 was considered statistically significant.

## Results

### Pentoxifylline Inhibits Bleomycin-Induced Pulmonary Fibrosis in Mice

**FIGURE 2:**
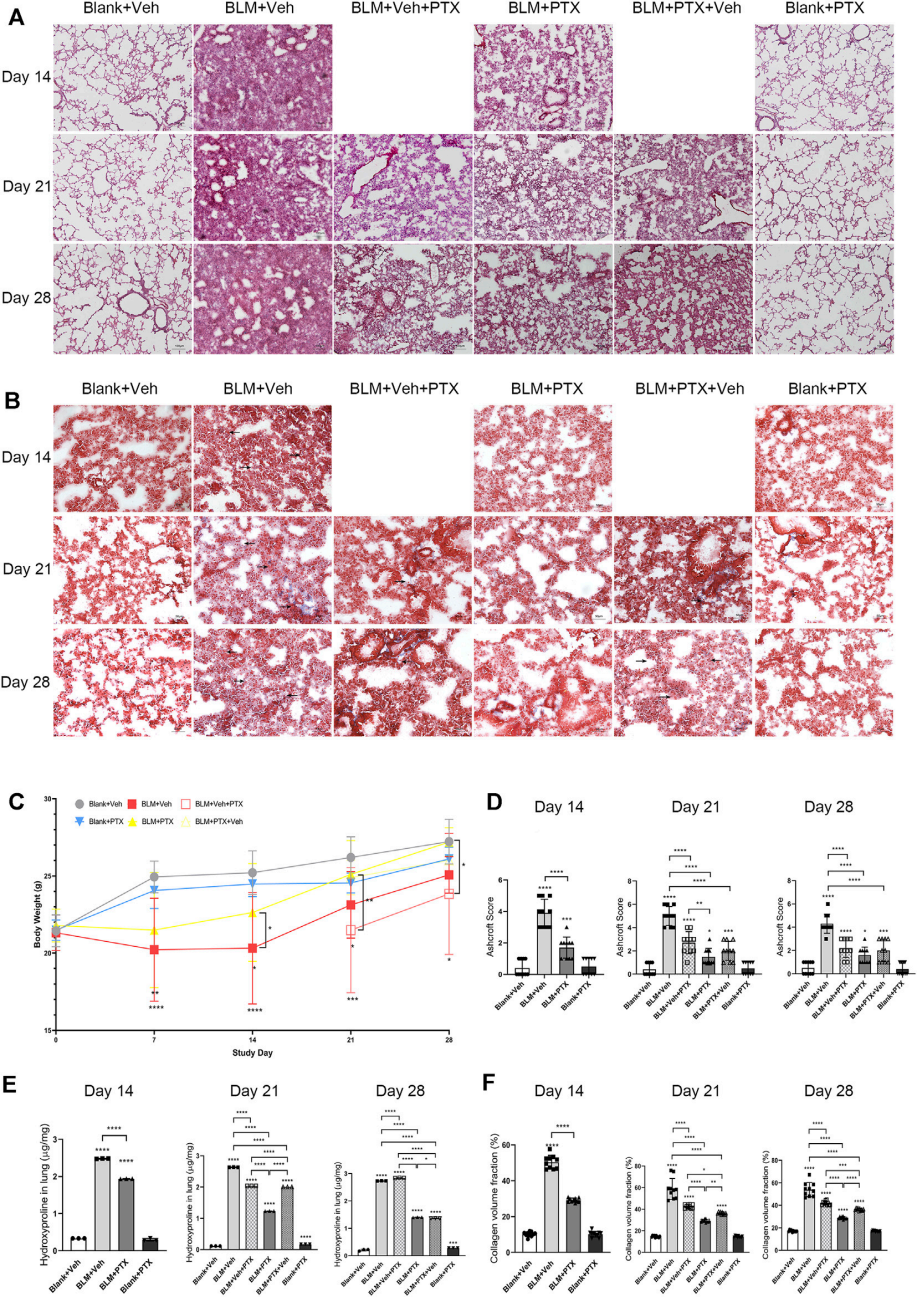
PTX inhibits the occurrence of pulmonary fibrosis induced by bleomycin in mice. **(A)** Representative microscopic results of hematoxylin-eosin (HE) staining in the frozen sections of mice lung tissue from Blank + Veh group, BLM + Veh group, BLM + Veh + PTX group, BLM + PTX group, BLM + PTX + Veh group, and Blank + PTX group on day 14, day 21, and day28 (*n* = 4 for each group). Original magnifications: ×100. Scale bars = 100 μm. **(B)** Representative microscopic results of Masson’s trichrome staining in the frozen sections of mice lung tissue in each group on day 14, day 21, and day 28 (*n* = 4 for each group). Original magnifications: ×200. Scale bars = 50 μm. Arrows indicate the pathological features of respective stains. **(C)** The mice body weight in each group on day 7, day 14, day 21, and day 28 (*n* = 4 for each group). **(D)** The Ashcroft score of mice lung tissue in each group on day 14, day 21, and day 28 (*n* = 10 for each group). **(E)** The hydroxyproline (HYP) content of the lung tissue from mice in each group on day 14, day 21, and day 28 (*n* = 3 for each group). **(F)** The collagen volume fraction (CVF) of mice lung tissue from each group on day 14, day 21, and day 28 (*n* = 10 for each group). Statistical significance is tested by one-way analysis of variance (ANOVA) with Tukey’s post-hoc test, *p < 0.05, **p < 0.01, ***p < 0.001, ****p < 0.0001.

We first tried to demonstrate that pentoxifylline (PTX) treatment suppressed the development of pulmonary fibrosis induced by bleomycin (BLM) in mice. Hematoxylin-eosin (HE) staining showed that BLM instillation induced severe alveolar lesions in mice, and the alveolar septum was prominently thickened and structurally disordered (Figure 2A). These pulmonary pathological changes already appeared on day 14, then peaked on day 21, and declined on day 28, which might be related to the self-limitation feature of the BLM-induced pulmonary fibrosis model. Meanwhile, Masson staining showed the deposition of enormous collagen fibers in the lung interstitium (Figure 2B). BLM instillation also significantly reduced mice’s body weight, suggesting that the physiological condition of mice was relatively poor (Figure 2C, Supplementary Figure S1). The Ashcroft score, hydroxyproline (HYP) content, and collagen volume fraction (CVF) of mice lung tissue further quantified the procession of fibrosis. BLM profoundly elevated these fibrosis indexes of lung tissue (Figures 2D–F). With the research progressing, CVF reached the highest point on day 21 and then decreased. Its overall fluctuation trend was consistent with the pathological changes in the lung tissue revealed by the HE staining and Masson staining (Figure 2F). The difference was that the HYP content soared on day 14 and remained high on day 21 and day 28 (Figure 2E).

Our study commenced PTX treatment on the second day after BLM instillation, once a day for 28 days. On day 14, day 21, and day 28, we observed that continuous PTX administration remarkably alleviated the alveolar lesions and collagen deposition in mice lung tissue (Figures 2A,B), accompanied by the decline of Ashcroft score, CVF, and HYP content in lung tissue (Figures 2D–F). Compared with the pulmonary fibrosis mice that received blank intervention simultaneously, PTX stopped the deterioration of pulmonary fibrosis during the study. However, the disorder of alveolar structure formed early still existed on day 28 (Figures 2A,B). In addition, PTX also contributed to putting on the body weight of mice and brought a stable improvement of the overall physical condition (Figure 2C).

### Pentoxifylline Downregulates the Expression of Fibrosis-Related Genes Both in Mice Lung Tissue and Pulmonary Fibroblasts

**FIGURE 3:**
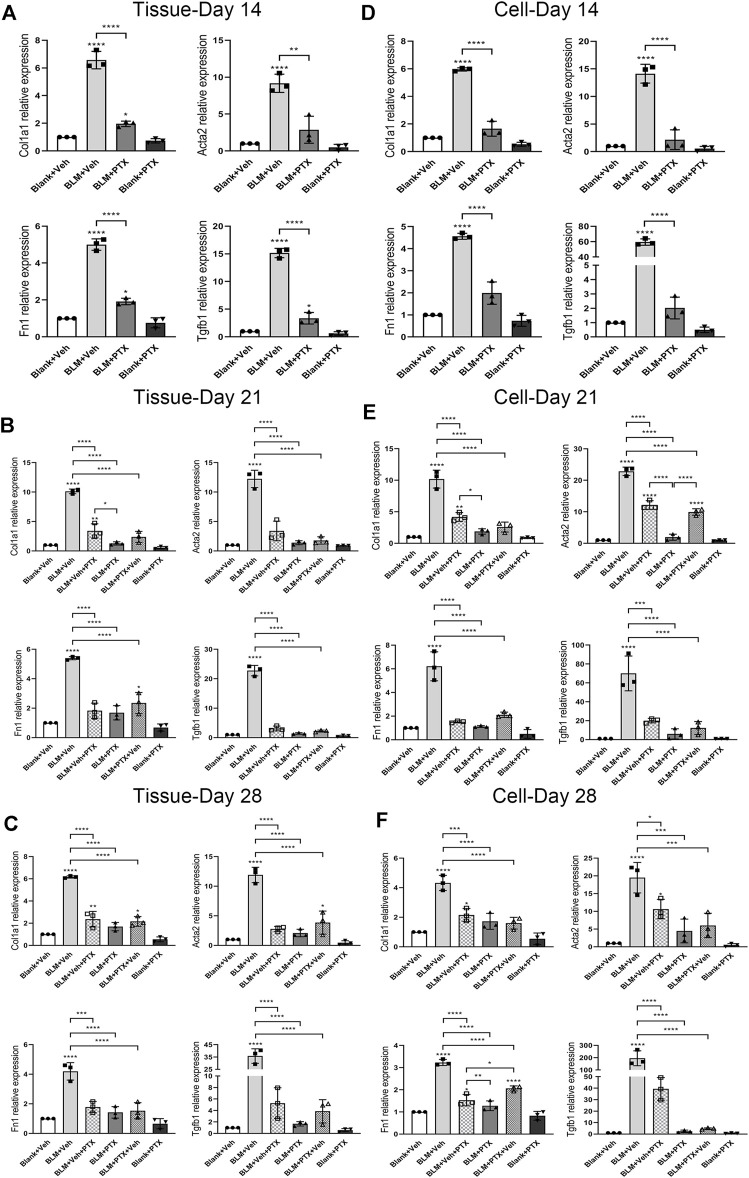
PTX therapy suppresses the expression of fibrosis-related genes. **(A)** Alpha-1 type I collagen (Col1a1), alpha-smooth muscle actin (Acta2), fibronectin 1 (Fn1), and transforming growth factor beta-1 (Tgfb1) gene expression levels in the mice lung tissue from Blank + Veh group, BLM + Veh group, BLM + PTX group, and Blank + PTX group on day 14 (*n* = 3 for each group). **(B,C)** Col1a1, Acta2, Fn1, Tgfb1 gene expression levels in the mice lung tissue from Blank + Veh group, BLM + Veh group, BLM + Veh + PTX group, BLM + PTX group, BLM + PTX + Veh group, and Blank + PTX group on day 21 and day 28 (*n* = 3 for each group). **(D–F)** The expression levels of Col1a1, Acta2, Fn1, Tgfb1 genes in the primary lung fibroblasts isolated from lung tissue of mice in each group on day 14, day 21, and day 28 (*n* = 3 for each group). Statistical significance is tested by one-way analysis of variance (ANOVA) with Tukey’s post-hoc test, *p < 0.05, **p < 0.01, ***p < 0.001, ****p < 0.0001.

Next, we conducted a series of real-time quantitative polymerase chain reaction (RT-qPCR) experiments to clarify the molecular details during the occurrence of pulmonary fibrosis and the PTX treatment. The expression of fibrosis-related genes alpha-1 type I collagen (Col1a1), alpha-smooth muscle actin (Acta2), fibronectin 1 (Fn1), and transforming growth factor beta-1 (Tgfb1) was all upregulated in the mice lung tissue on day 14 after BLM instillation (Figures 3A–C). While the increase of Col1a1 gene expression peaked at day 21 and retracted on day 28, the upregulation of Acta2, Fn1, and Tgfb1 genes existed until day 21 and day 28, and further with a consistent upward trend.

PTX could significantly inhibit the ascending expression of these fibrosis-related genes triggered by BLM in mice lung tissue (Figures 3A–C). The continuous administration of PTX lowered the expression of Col1a1, Acta2, Fn1, and Tgfb1 genes in lung tissue on day 14 back close to their baseline (Figure 3A). Moreover, with the PTX treatment extended to day 21 and day 28, the suppression of these gene expressions became even more pronounced (Figures 3B,C).

Besides, the *in vivo* effects of BLM instillation and PTX administration on the expression level of fibrosis-related genes in mouse primary lung fibroblasts had similar features compared with that in lung tissue, suggesting that lung fibroblasts attached an essential and positive relationship on the progression of pulmonary fibrosis (Figures 3D–F). Col1a1, Acta2, Fn1, and Tgfb1 genes were consistently raised in mouse primary lung fibroblasts on day 14, day 21, and day 28 after BLM instillation (Figures 3D–F). Acta2 and Fn1 genes in lung fibroblasts had more marked enhancements than their expression levels in lung tissue, while the Col1a1 and Fn1 gene expression variations were similar to that in lung tissue (Figures 3D–F). The administration of PTX returned the intracellular levels of these fibrosis-related genes at different time points all back to around their basal level, which corresponded to the anti-fibrotic effect of PTX in the lung tissue but was in a more intense way (Figures 3D–F).

### Changing Treatment Timing of Pentoxifylline has Impacts on its Anti-Fibrotic Effectiveness

We further studied the substantial impact of the treatment timing on the anti-fibrotic effect of PTX. As we mentioned above, BLM already led to noticeable pulmonary lesions on day 14. Even if mice commenced PTX therapy on day 15, the performance of alveolar lesions and fibrotic indexes in mice lung tissue could still be effectively ameliorated by PTX on day 21 and day 28 (Figures 2A,B,D–F). The PTX treatment starting on day 15 after the BLM instillation could still significantly suppress the intrapulmonary expression of these fibrosis-related genes. There was almost no noticeable difference compared with mice that started PTX treatment earlier on day 1 (Figures 3B,C). However, when persistent PTX treatment for 14 days had suspended BLM-induced pulmonary fibrosis, removing PTX treatment resulted in the slight rekindling of alveolar lesions and collagen deposition in lung tissue, but there was no obvious abnormality in body weight (Figures 2A–D). In addition, PTX still acted as a persistent disincentive to pulmonary fibrosis progression after mice stopped PTX treatment on day 14, with only Col1a1 and Acta2 genes showing slight recovery on day 28 (Figures 3B,C). It could be seen that a period of PTX therapy, though it might not be early or sustained enough, could benefit mice in various degrees, no matter in pulmonary lesions or overall physiological conditions.

Moreover, mouse primary lung fibroblasts were more sensitive to the alteration of PTX administration timing (Figures 3D–F). Delaying the initiation of PTX treatment did not decrease its suppressive effect on fibrosis progression in lung fibroblasts, but it might indeed take longer to benefit mice. This influence was particularly evident in the intracellular expression of Col1a1 and Acta2 genes on day 21 (Figures 3D–F). Meanwhile, quitting the PTX treatment in advance led to a noticeable rebound in the intracellular levels of fibrosis-related genes, especially the Acta2 gene on day 21 and the Fn1 gene on day 28 (Figures 3D–F). In general, the minor differences in mice lung tissue produced by PTX treatment of different timing and duration would be sensitively amplified in the pulmonary fibroblasts isolated from the tissue. The sustained anti-fibrotic effect of PTX was not much influenced by the timing of administration when the drug dose remained.

### Pentoxifylline Inhibits Bleomycin-Induced Lung Senescence in Mice

**FIGURE 4:**
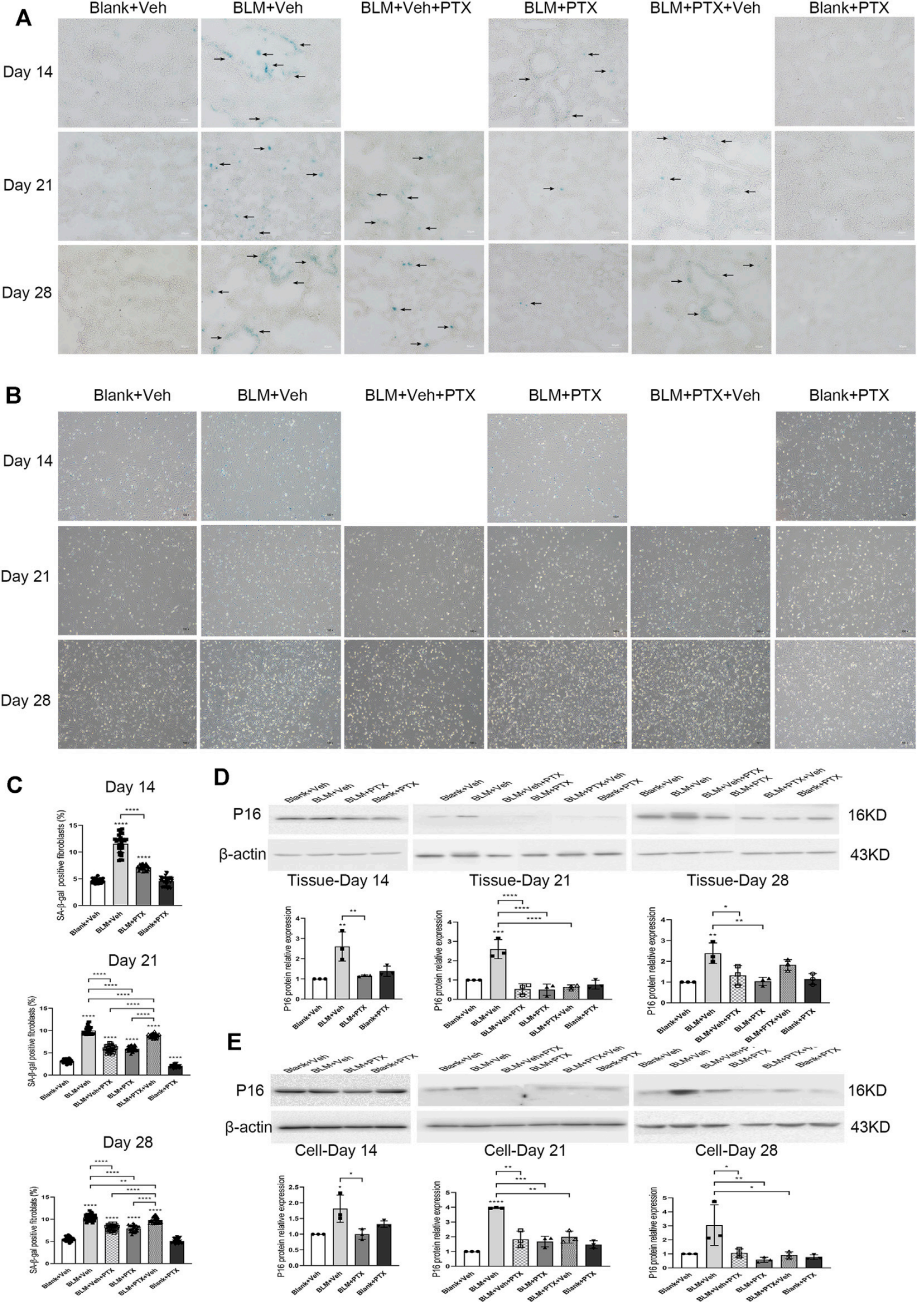
PTX inhibits the formation of cellular senescence induced by bleomycin (BLM) in mice. **(A)** Representative microscopic results of senescence-associated β-galactosidase (SA-β-gal) staining in the frozen sections of mice lung tissue from Blank + Veh group, BLM + Veh group, BLM + Veh + PTX group, BLM + PTX group, BLM + PTX + Veh group, and Blank + PTX group on day 14, day 21, and day 28. Original magnifications: ×200. Scale bars = 50 μm. Arrows indicate the pathological features of respective stains (*n* = 4 for each group). **(B,C)** Representative microscopic results and the positive rate of SA-β-gal staining in the primary lung fibroblasts isolated from lung tissue of mice in each group on day 14, day 21, and day 28 (*n* = 4 for each group). Original magnifications: ×100. **(D)** The expression level of p16 protein in the lung tissue from mice in each group on day 14, day 21, and day 28 (*n* = 3 for each group). **(E)** The expression level of p16 protein in the primary lung fibroblasts isolated from lung tissue of mice in each group on day 14, day 21, and day 28 (*n* = 3 for each group). Statistical significance is tested by one-way analysis of variance (ANOVA) with Tukey’s post-hoc test, *p < 0.05, **p < 0.01, ***p < 0.001, ****p < 0.0001.

Previous studies made us believe that cellular senescence plays a central role in the development and progression of fibrous lung diseases. Therefore, we next tried to probe into whether the PTX administration also had a connection with senescence resistance. We conducted senescence-associated β-galactosidase (SA-β-gal) staining, specific enzyme histochemistry staining for senescent cells, on frozen sections of mice lung tissue (Figure 4A). The results of the SA-β-gal staining illustrated that BLM instillation induced the accumulation of strong positive signals in the lung tissue on day 14, especially in lung interstitium, manifested as the dark blue staining results sharply contrast with the transparent or translucent normal lung tissue under the microscope (Figure 4A). The positive markers of SA-β-gal staining were more widely distributed in mice lung tissue on day 21, and on day 28, the intensity and breadth of positive signals tended to decline. In other words, BLM instillation caused severe lesions and fibrosis in the lung tissue, as well as cellular senescence. In addition, at these three different time points after BLM instillation, the primary lung fibroblasts isolated from mouse lung tissue showed a significantly increased positive rate of SA-β-gal staining (Figures 4B,C). It was also proved that the expression level of p16 protein stably remained at a high level on day 14, day 21, and day 28 after BLM instillation, both in mice lung tissue and primary pulmonary fibroblasts (Figures 4D,E).

PTX successfully blocked the occurrence of lung senescence induced by BLM in mice. SA-β-gal staining results of lung tissue on day 14, day 21, and day 28 after PTX therapy showed that continuous PTX administration could clear almost all senescence-associated blue positive signals in lung tissue, as well as p16 protein expression elevated by BLM (Figures 4A,D,E). We then tried to determine whether pulmonary fibroblasts are affected during these changes. SA-β-gal staining illustrated that there is also a marked drop in the positive rates of primary pulmonary fibroblasts isolated from mouse lungs (Figures 4B,C). Although the positive rate did not return to their basic level showed in the fibroblasts from control group mice, the expression level of p16 protein in these fibroblasts was significantly reduced to the basic level (Figure 4E). In other words, removing the senescent phenotype of lung fibroblasts was part of the anti-senescence effect of PTX administration.

### Pentoxifylline Downregulates the Expression of Senescence-Related Genes Both in Mice Lung Tissue and Pulmonary Fibroblasts

**FIGURE 5:**
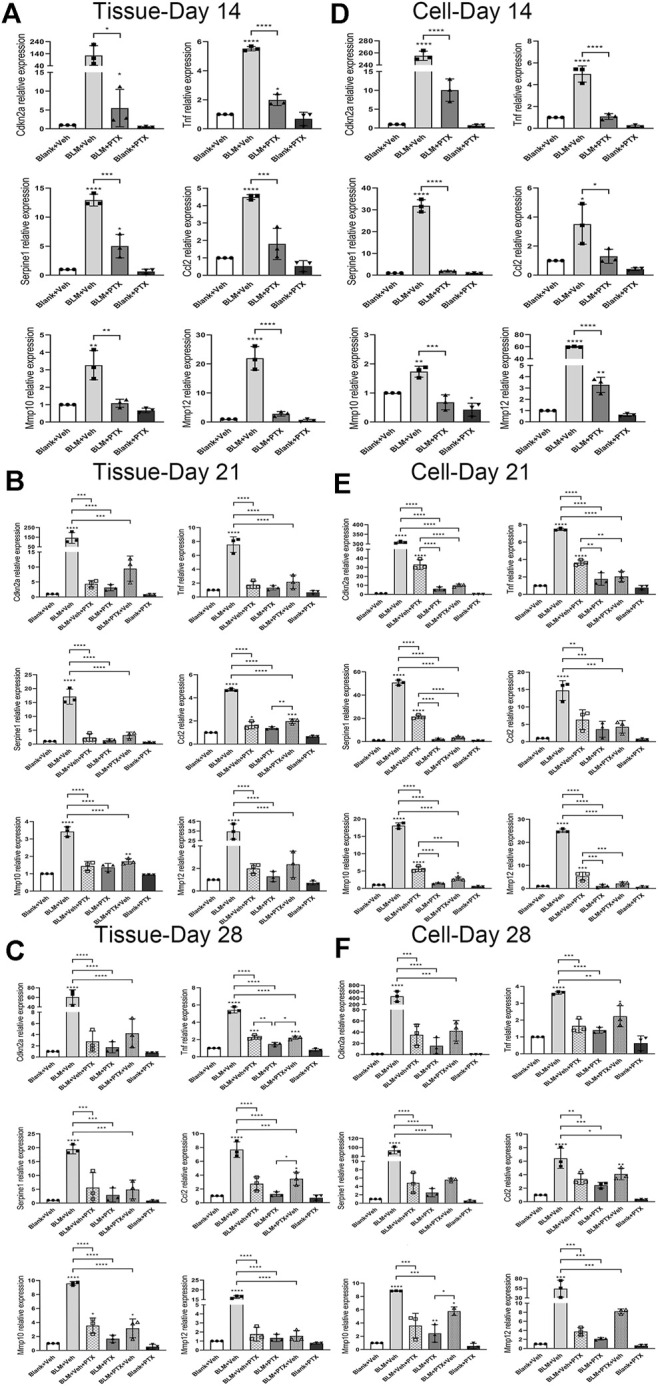
PTX therapy suppresses the expression of senescence-related genes. **(A)** The expression levels of cyclin dependent kinase inhibitor 2a (Cdkn2a) gene encodes p16 protein, which dominates the occurrence of cellular senescence. Tumor necrosis factor (Tnf), serpin family E member 1 (Serpine1), C-C motif chemokine ligand 2 (Ccl2), matrix metallopeptidase 10 (Mmp10), and matrix metallopeptidase 12 (Mmp12) genes in the mice lung tissue from Blank + Veh group, BLM + Veh group, BLM + PTX group, and Blank + PTX group on day 14 (*n* = 3 for each group). **(B,C)** The expression levels of Cdkn2a, Tnf, Serpine1, Ccl2, Mmp10, and Mmp12 genes in the mice lung tissue from Blank + Veh group, BLM + Veh group, BLM + Veh + PTX group, BLM + PTX group, BLM + PTX + Veh group, and Blank + PTX group on day 21 and day 28 (*n* = 3 for each group). **(D–F)** The expression levels of Cdkn2a, Tnf, Serpine1, Ccl2, Mmp10, Mmp12 genes in the primary lung fibroblasts isolated from lung tissue of mice in each group on day 14, day 21, and day 28 (*n* = 3 for each group). Statistical significance is tested by one-way analysis of variance (ANOVA) with Tukey’s post-hoc test, *p < 0.05, **p < 0.01, ***p < 0.001, ****p < 0.0001.

We performed another set of RT-qPCR experiments to explore how the senescence-related gene expressions were regulated during the PTX therapy and how much the fibroblast senescence participated in the lung senescence. Cyclin-dependent kinase inhibitor 2a (Cdkn2a) gene encodes p16 protein, which dominates the occurrence of cellular senescence. Tumor necrosis factor (Tnf), serpin family E member 1 (Serpine1), C-C motif chemokine ligand 2 (Ccl2), matrix metallopeptidase 10 (Mmp10), and matrix metallopeptidase 12 (Mmp12) genes are involved in encoding the central components of senescence-associated secretory phenotype (SASP), which is one of the cellular senescence biomarkers. Cdkn2a gene expression in lung tissue was prominently upregulated by BLM instillation. It was raised over 200 fold on day 14 and day 21 and decreased on day 28 (Figures 5A–C). BLM also elevated the expression of SASP-related genes in lung tissue. The varying degrees of these genes were generally lower than that of the Cdkn2a gene, and their increasing range was also different from each other. And they all showed a similar fluctuation trend with the Cdkn2a gene during the study period. The ascending of these senescence-related genes in lung tissue induced by BLM could be well suppressed by PTX (Figures 5A–C). On day 14, Cdkn2a, Tnf, and Serpine1 gene expressions did not return to the baseline level under the treatment of PTX (Figure 5A). However, as the study went on to day 21 and day 28, PTX maintained the expression of these senescence-related genes in lung tissue at a regular and low level (Figures 5B,C). Besides, senescence-related genes expression was affected more at the lung fibroblast level than at the lung tissue level (Figures 5D–F). BLM increased Cdkn2a, Serpine1, and Mmp12 genes expression in lung fibroblasts on day 14, day 21, and day 28. The intracellular expression of Tnf, Ccl2, and Mmp10 genes was also upregulated by BLM instillation and declined on day 28 (Figures 5A–F). The increasing relevant multiples of all these senescence-related genes in pulmonary fibroblasts induced by BLM instillation were much more than those in lung tissue. And as always, PTX administration significantly downregulated the expression of senescence-related genes in lung fibroblasts, consistent with its curative effect against senescence in lung tissue (Figures 5D–F).

### Changing Treatment Timing of Pentoxifylline has Impacts on its Anti-Senescence Effectiveness

The treatment timing could also influence the anti-senescence effect of PTX. PTX administration beginning on day 15 still removed most senescent cells from lung tissue on day 21 and day 28. The clearance of senescent cells was partly illustrated by the decreased positive markers of SA-β-gal staining in lung tissue and the decreased positive rate of SA-β-gal staining in primary lung fibroblasts (Figures 4A,B). However, stopping PTX treatment on day 14 caused a pronounced rebound in senescent markers. Whether in lung tissue or fibroblasts, once PTX treatment was discontinued, the positive markers of SA-β-gal staining re-aggregated rapidly and led to remarkable changes widespread in lung tissue (Figures 4A,B). And when we focused on the effect of PTX administration on the p16 protein level in lung tissue and fibroblasts, the results were still optimistic (Figures 4C,D). Delayed administration and early withdrawal of PTX did not significantly alter the expression level of p16 protein both in lung tissue and lung fibroblasts (Figures 4C,D). We had to say that the inhibitory effect of PTX on senescence in mice was similar but different at the level of lung tissue and fibroblasts, possibly because of the large variety of cells in the lung tissue with various functions and different reactions to PTX therapy.

The expressions of senescence-related genes told us more molecular details. If PTX treatment began on day 15 after BLM instillation, although the anti-senescence effect of PTX in lung tissue did not disappear, the effect did suffer from a weakening (Figures 5B,C). The diminished impact after delayed PTX treatment was found in the expression alteration of senescence-related genes, which was mainly reflected in the expressional changes of Ccl2, Tnf, and Mmp10 genes (Figures 5B,C). Compared with the performance of PTX treatment in lung tissue, PTX took a longer time to start inhibiting the expression of these genes in fibroblasts, which was specifically manifested in that PTX treatment commenced on day 15 did not repress the expression of these genes to a satisfactorily low level on day 21 except the Ccl2 gene (Figure 5E). It was until day 28 that PTX therapy accomplished this goal (Figure 5F).

What is more, once PTX administration stopped on day 14, Ccl2 and Mmp10 gene expressions in lung tissue immediately showed sensitive and rapid recovery on day 21. On day 28, the Tnf gene also joined the rebounding team (Figures 5B,C). On the other hand, the influence on the senescence-related genes in lung fibroblasts caused by halting PTX administration in advance was not as apparent as that in lung tissue, and only the Mmp10 gene slightly increased its intracellular level after drug withdrawal (Figures 5D–F). The failure to maintain the anti-senescence effect in lung tissue after discontinuance of PTX intervention might be due to other types of cells in the lung. And these also suggested that pulmonary fibroblasts were not the only type of cell influenced by PTX administration in the BLM-induced mice model.

### Long-Term Administration of Pentoxifylline Successively Benefits Mice in the Remission of Pulmonary Fibrosis

**FIGURE 6:**
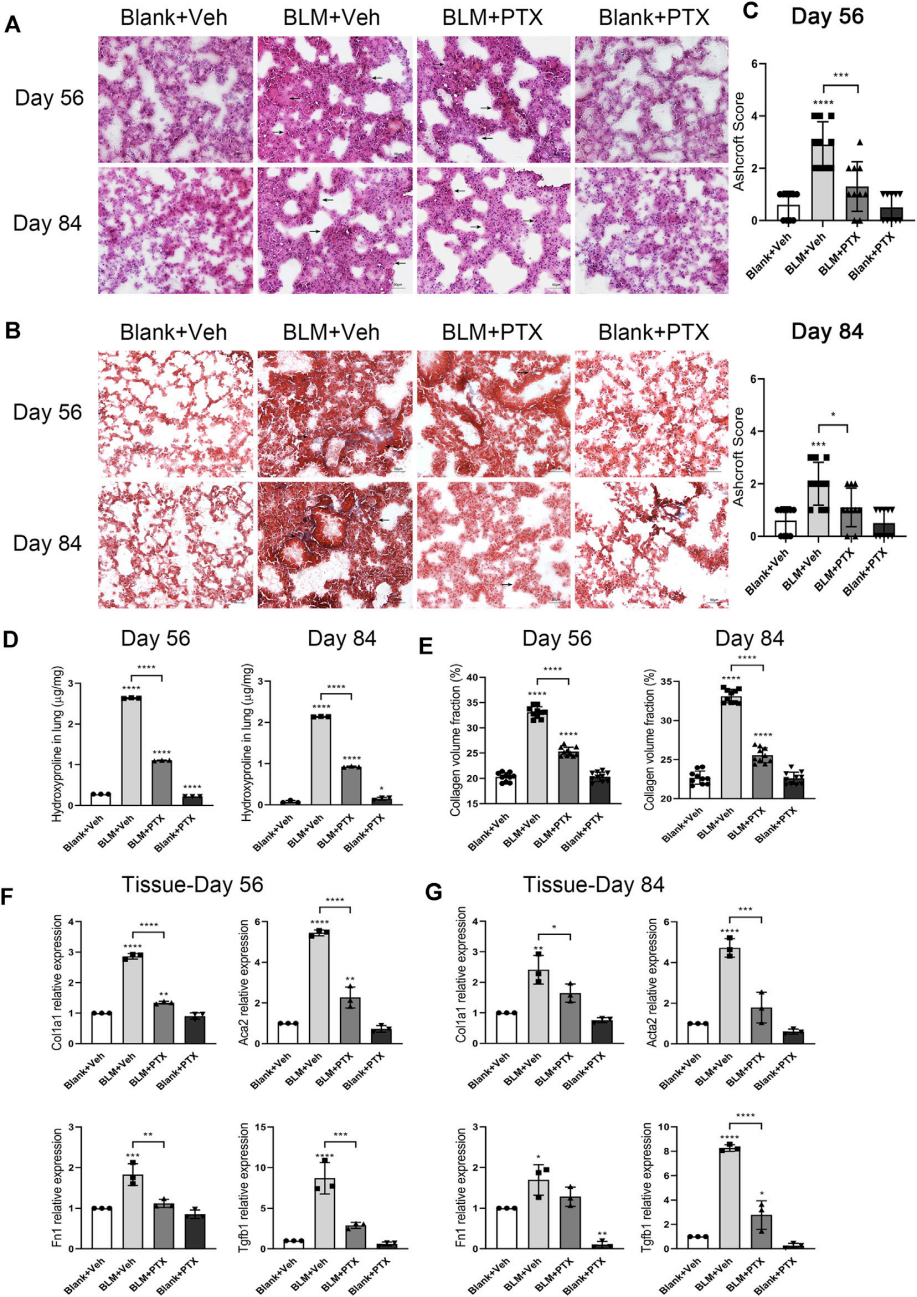
Long-term PTX administration affects the lung fibrosis remission in mice. **(A,B)** Representative microscopic results of HE staining and Masson staining in the frozen sections of mice lung tissue from Blank + Veh group, BLM + Veh group, BLM + PTX group, and Blank + PTX group on day 56 and day 84 (*n* = 4 for each group). Original magnifications: ×200. Scale bars = 50 μm. Arrows indicate the extent of pathological features of respective stains. **(C)** The Ashcroft score of mice lung tissue in each group on day 56 and day 84 (*n* = 10 for each group). **(D)** The HYP content of the lung tissues from mice in each group on day 56 and day 84 (*n* = 3 for each group). **(E)** The CVF of mice lung tissue from each group on day 56 and day 84 (*n* = 10 for each group). **(F,G)** The expression levels of Col1a1, Acta2, Fn1, Tgfb1 genes in the lung tissue from mice in each group on day 56 and day 84 (*n* = 3 for each group). Statistical significance is tested by one-way analysis of variance (ANOVA) with Tukey’s post-hoc test, *p < 0.05, **p < 0.01, ***p < 0.001, ****p < 0.0001.

To explore whether the prolonged administration of PTX could produce long-term benefits about their anti-fibrosis effect, we continued to treat the remaining mice in each group with the same dose of PTX or vehicle. HE staining showed that, compared with the mice on day 28, the alveolar damage and fibrosis in lung tissue induced by BLM continued to alleviate on day 56 and day 84 (Figure 6A). With the consistent assistance of PTX treatment, pulmonary lesions in mice acquired a better recovery rate. Masson staining also intuitively reflected this recovery through reduced collagen deposition in mice lung tissue (Figure 6B). As the quantitative standards of pulmonary fibrosis, Ashcroft score, HYP content, and CVF in mice lung tissue also reflected the consistent results (Figures 6C–E). On day 56 and day 84, the values of these indexes in the PTX-treated mice continuously decreased at a quicker pace compared with the mice receiving blank treatment, which reflected the accelerating recovery of fibrosis under the sustained PTX administration. On the other hand, with the ongoing research, the Ashcroft score, HYP content, and CVF in mice lung tissue from negative control were slightly raised, suggesting that mice would show fibrous lung performance with aging (Figures 6C–E).

We further studied the expression levels of fibrosis-related genes in mice lung tissue on day 56 and day 84, trying to obtain a more accurate and intuitive explanation about the anti-fibrosis effect of successive PTX administration (Figures 6F,G). On day 56, the expression of Col1a1, Acta2, Fn1, and Tgfb1 genes in mice lung tissue was still upregulated because of BLM instillation early on day 0, but it was far less than that on day 14, day 21, or day 28 (Figure 6F). The expression levels of these fibrosis-related genes in lung tissue continued to decline on day 84, and their changing degree was relatively minor, except for the Tgfb1 gene (Figure 6G).

Moreover, although the anti-fibrosis effect of PTX was remarkable while the study moved on to day 56 and day 84, there was still some instability. On day 56, PTX administration failed to reduce the expression of Col1a1 and Acta2 genes to their baseline levels in lung tissue. And on day 84, the same thing happened to the intrapulmonary expression level of the Fn1 gene (Figures 6F,G). These might be associated with the imbalance between the curative effects from PTX and the aging-associated fibrotic changes in mice. And the self-recovery of the BLM-induced pulmonary fibrosis model made the suppressive effects of PTX administration seem to be less evident (Figures 6F,G).

### Long-Term Administration of Pentoxifylline Consistently Benefits Mice in the Delaying Occurrence of Cellular Senescence

**FIGURE 7:**
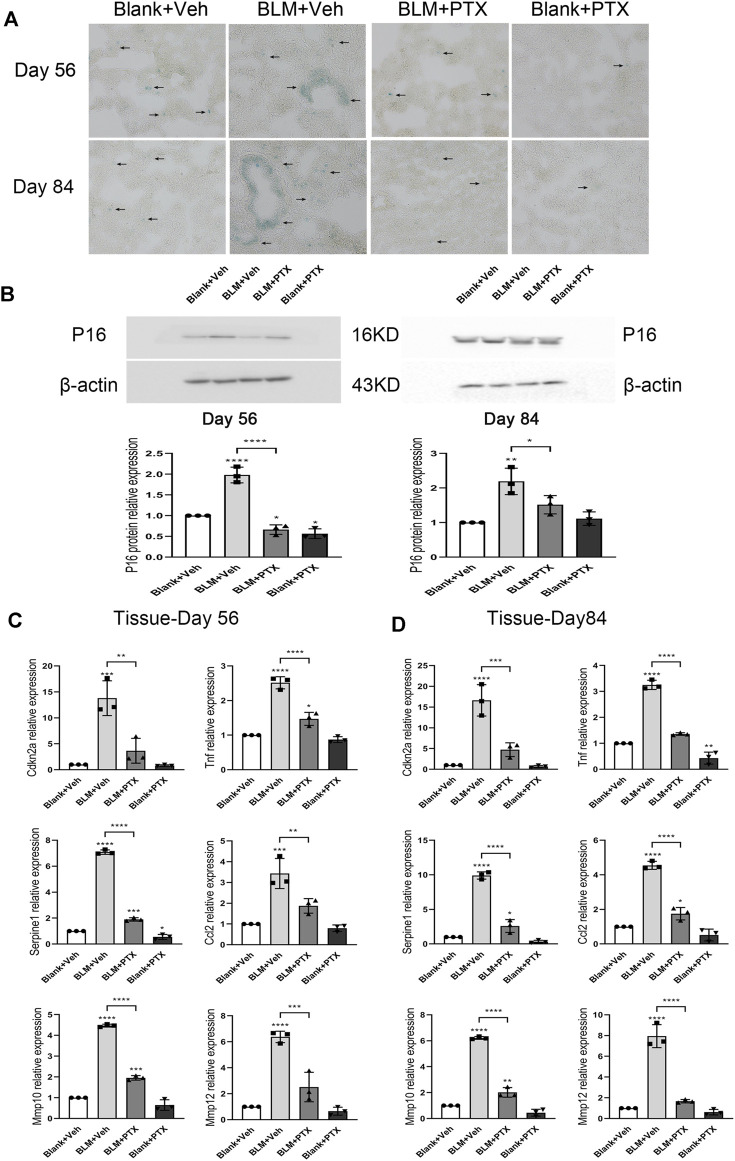
Long-term PTX administration influences the cellular senescence development in mice. **(A)** Representative microscopic results of SA-β-gal staining in the frozen sections of mice lung tissue from Blank + Veh group, BLM + Veh group, BLM + PTX group, and Blank + PTX group on day 56 and day 84 (*n* = 4 for each group). Original magnifications: ×200. Scale bars = 50 μm. Arrows indicate the extent of pathological features of respective stains. **(B)** The expression level of p16 protein in the lung tissue from mice in each group on day 56 and day 84 (*n* = 3 for each group). **(C,D)** The expression levels of Cdkn2a, Tnf, Serpine1, Ccl2, Mmp10, Mmp12 genes in the lung tissue from mice in each group on day 56 and day 84 (*n* = 3 for each group). Statistical significance is tested by one-way analysis of variance (ANOVA) with Tukey’s post-hoc test, *p < 0.05, **p < 0.01, ***p < 0.001, ****p < 0.0001.

On day 56 and day 84 of the whole study process, the mice used in our research were transitioning from mature individuals to more elderly individuals. We further paid attention to whether the sustained administration of PTX benefits mice in the perspective of senescence resistance. The senescence conditions of mice lung tissue on day 56 and day 84 were illustrated by SA-β-gal staining (Figure 7A). At these two time points, positive signals of SA-β-gal staining still gathered in the lung interstitium. The intensity of positive SA-β-gal staining signals was weaker on day 56 and day 84 than on day 28, and the dark blue markers in the lung tissue began to appear in a broader range (Figure 7A). In particular, sporadic positive signals of SA-β-gal staining successively appeared in the lung tissue of mice in the negative control group (Figure 7A). The PTX administration continued till day 84 suppressed the lung senescence development in mice during its medication. Remained minor positive signals of SA-β-gal staining suggested that PTX administration did not entirely block the occurrence of senescence (Figure 7A). In addition, the expression level of p16 protein in mice lung tissue on day 56 and day 84 after BLM instillation was still slightly higher than the baseline and was similar to its expression level on day 28 (Figure 7B). The sustained PTX therapy, as always, fully decreased the expression of p16 protein in the lung tissue.

Senescence-related genes also sensitively reflected the changes in mice lung tissue on day 56 and day 84 (Figures 7C,D). When the study was carried out till day 56 and day 84, the BLM instillation on day 0 no longer seemed to play an essential role in promoting lung senescence but was replaced by the effect of age growth from mice themselves. Specifically, the expression levels of Cdkn2a, Tnf, Serpine1, Ccl2, Mmp10, and Mmp12 genes in lung tissue on day 56 were significantly lower than those on day 28 (Figure 7C) and were also lower than those on day 84 (Figure 7D). And their ascending on day 84 was not as remarkable as their elevation in the mice’s lung tissue on day 28. Based on these changes, long-term PTX administration significantly inhibited the expression of these senescence-related genes in lung tissue, but the actual effect was not as impressive as it performed on day 28 (Figures 7C,D). On day 56, the intrapulmonary expression levels of Tnf and Mmp10 genes were not fully downregulated by PTX. On day 84, PTX did not completely inhibit the upregulated expression of Serpine1, Ccl2, and Mmp10 genes (Figures 7C,D). Overall, through long-term PTX administration, mice could simultaneously acquire the benefits of anti-senescence at the macro and gene expression levels.

### Pentoxifylline Inhibits Cellular Senescence and Relevant Fibrogenesis in Human Fibroblasts

**FIGURE 8:**
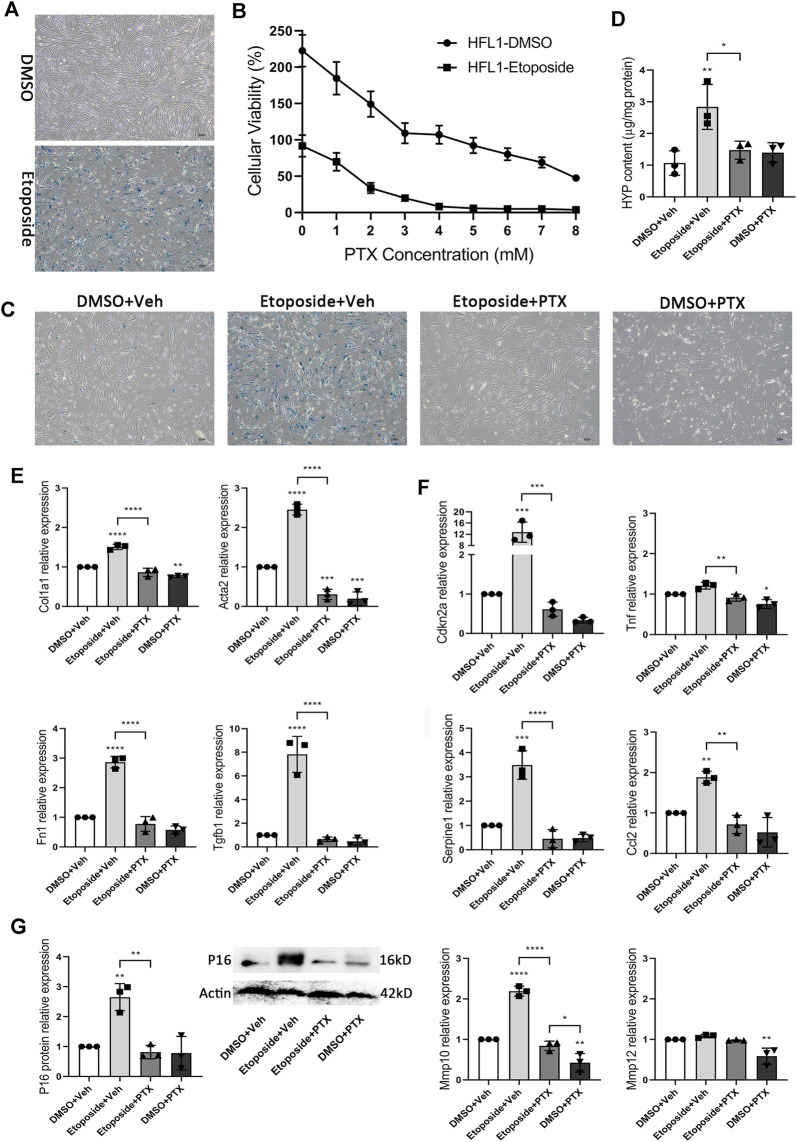
PTX inhibits cellular senescence and relevant fibrogenesis in HFL1 cells. **(A)** Representative microscopic results of SA-β-gal staining in DMSO or Etoposide-exposed HFL1 cells (*n* = 3 for each group). Original magnifications: ×100. **(B)** Cellular viability of DMSO or Etoposide-exposed HFL1 cells after 24 h PTX treatment, and is indicated as a percentage of plating density at 0 h of treatment (*n* = 3 for each group). **(C)** Representative microscopic results of SA-β-gal staining in DMSO or Etoposide-exposed HFL1 cells after 24 h PTX or Vehicle treatment (*n* = 3 for each group). Original magnifications: ×100. **(D)** The HYP content of cellular supernatants from HFL1 cells in each group (*n* = 3 for each group). **(E)** The expression levels of Col1a1, Acta2, Fn1, Tgfb1 genes in the HFL1 cells in each group (*n* = 3 for each group). **(F)** The expression levels of Cdkn2a, Tnf, Serpine1, Ccl2, Mmp10, Mmp12 genes in the HFL1 cells in each group (*n* = 3 for each group). **(G)** The expression level of p16 protein in DMSO or Etoposide-exposed HFL1 cells after 24 h PTX or Vehicle treatment (*n* = 3 for each group). Statistical significance is tested by one-way analysis of variance (ANOVA) with Tukey’s post-hoc test, *p < 0.05, **p < 0.01, ***p < 0.001, ****p < 0.0001.

In the study above, we had confirmed the effectiveness of PTX treatment in animal experiments. We also needed to confirm that these therapeutic effects were still practical when the species background of the drug was human. Therefore, we chose human fetal lung fibroblast HFL1 as our research object. HFL1 cells treated with etoposide were positively stained by SA-β-gal staining, suggesting that the HFL1 cell aging model was successfully constructed (Figure 8A). CCK8 experiment showed that the survival rates of HFL1 cells exposed to DMSO or etoposide were significantly different between the two groups 24 h after receiving different concentrations of PTX (Figure 8B). We hoped to find a suitable concentration to make PTX have enough lethality to senescent cells, but the toxic effect on normal cells is acceptable. When the concentration of PTX administration was 4 mM, most of the etoposide-exposed HFL1 cells died, but the number of DMSO-exposed HFL1 cells remained roughly stable (Figure 8B). SA-β-gal staining also indicated that the viable senescent HFL1 cells after PTX treatment at a concentration of 4 mM for 24 h showed decreased positive signs of SA-β-gal staining, suggesting the scavenging effect of PTX on senescent HFL1 cells (Figure 8C). More details of PTX treatment were reflected in the expression of senescence-related genes. In HFL1 cells exposed to etoposide, the expression of Cdkn2a, Serpine1, Ccl2, and Mmp10 genes was significantly upregulated. The expression level of the P16 protein also changed significantly, which was similar to the relevant experimental results in mice. However, we did not observe the increased expression levels of Tnf and Mmp12 genes, which might be because the composition of SASP varied with the change of cell type and cell state (Figures 8F,G). In senescent HFL1 cells receiving PTX administration, the expression levels of these senescence-associated genes decreased to a level similar to that of normal HFL1 cells, suggesting the anti-senescence effect of PTX treatment (Figures 8F,G).

In addition, the HYP content in the cellular supernatants of etoposide-treated HFL1 cells was significantly higher than that of DMSO-treated HFL1 cells, and the intracellular expression of fibrosis-related genes Col1a1, Acta2, Fn1, and Tgfb1 were also significantly upregulated. In the aging process, HFL1 cells also performed the formation of fibrosis, and PTX treatment significantly inhibited the occurrence of senescence-related fibrogenesis (Figures 8D,E). Under PTX treatment, the production of HYP and the expression level of fibrosis-related genes in senescence HFL1 cells were maintained at a low level similar to that in the negative control group (Figures 8D,E). Overall, PTX showed excellent anti-senescence and anti-fibrosis effects in human HFL1 cells.

## Discussion

In this study, pentoxifylline (PTX) treatment was used to inhibit the pulmonary fibrosis formation in bleomycin (BLM)-injured mice. Since the development of cellular senescence could also be observed in the lung tissue of BLM-injured mice, we further evaluated the anti-senescence effects of PTX in mice. PTX treatment exhibited potent anti-inflammatory, anti-fibrotic, and anti-aging effects in mice. This study was not the first time PTX was used to treat animals with BLM-induced lung injury. Our experiments extended the longitudinal timeline and further explored the role of fibroblast senescence in the formation of lung injury and pulmonary fibrosis (Entzian et al., 1997; Entzian et al., 1998).

Mice that did not receive PTX treatment experienced significant weight loss on day 7 after BLM administration (Figure 2C). On day 14, severe alveolar lesions and fibrosis were observed in the lung tissue of these mice, accompanied by tangible signs of cellular senescence such as positive senescence-associated β-galactosidase (SA-β-gal) staining and high expression level of p16 protein (Figures 2, 4). Meanwhile, gene expressions related to fibrosis and senescence were significantly upregulated in lung tissue and primary lung fibroblasts after BLM instillation (Figures 3, 5). Fibrosis-related genes include Col1a1, Acta2, Fn1, and Tgfb1 gene, and senescence-related genes composed of Cdkn2a, Tnf, Serpine1, Ccl2, Mmp10, and Mmp12 gene, all of which reflected the intensity of lung fibrosis and senescence events in lung tissue and pulmonary fibroblasts. The results illustrated that both pulmonary fibrosis and senescence caused by BLM modeling were stably ameliorated by PTX treatment (Figures 2, 4). PTX administration effectively stopped the progression of pulmonary fibrosis and lung senescence; meanwhile, fibrosis-related genes and senescence-related genes were downregulated by PTX in the lung tissue and fibroblasts in various degrees (Figures 3, 5). In most cases, the expression of these genes was affected more in the primary lung fibroblasts, suggesting that other cells in lung tissue might play the opposite role to pulmonary fibroblasts in the development of pulmonary fibrous disease and PTX treatment. We needed further researches to illustrate the specific role of immune cells, epithelial cells, or endothelial cells in the processes of PTX treatment, which might be accomplished with the help of cell sorting technology in subsequent studies.

We started PTX treatment on the next day of BLM administration in the experiment, but such early and timely interventions were scarce for clinical patients with IPF. For IPF patients, reasons for medical visits were usually respiratory symptoms such as chest tightness and shortness of breath after daily activity (Raghu et al., 2011). The main diagnostic criteria of IPF include abnormal pulmonary function results and bilateral HRCT lung imaging, which also indicates that a suspected IPF patient has already developed a certain extent of physiological function changes. Therefore, we should consider that the effectiveness of PTX treatment might be affected by its treatment timing. It is generally believed that the pulmonary lesions induced by BLM instillation were mainly inflammatory changes in the first 14 days, and gradually changed to fibrous changes afterward, day 14 after BLM administration was used as the time point to start or stop PTX therapy in each group of mice to simulate the treatment timing that often occurs in clinical practice. PTX treatment commenced on day 15 did not obviously affect the therapeutic effects of PTX in the mice lung tissue. But in primary lung fibroblasts, PTX took longer to restore the expression of fibrosis-related genes and senescence-related genes to their baseline level (Figures 2–5). Once the PTX administration was stopped, lung fibroblasts quite sensitively showed a rebound in the expression of genes related to fibrosis and senescence (Figures 2–5). And with a certain delay, signs related to fibrosis and cellular senescence reappeared in the lung tissue after stopping PTX therapy on day 14, suggesting that lung fibrosis and senescence formation seemed to be the result of gradual accumulation. However, as in earlier studies, the intrapulmonary lesions of mice resulting from BLM instillation peaked on day 21 and began to show spontaneous remission on day 28. In other words, after day 28, it was difficult to tell whether the recovery of lung lesions in mice was due to the beginning of PTX treatment or the self-limitation of the BLM mice model. Hence our study about the PTX treatment timing halted on day 28 after BLM administration.

Instead, we extended the duration of PTX treatment for BLM-injured mice to 56 days or 84 days, trying to determine the further therapeutic effects of a long-term PTX administration by comparing the recovery pace of pulmonary fibrosis in mice from different groups. On day 56 and day 84, we could observe the better remission of pulmonary fibrosis in mice that receive PTX therapy, which indicated the sustained anti-fibrosis effects of the long-term PTX administration (Figure 6). And since mice were gradually moving to the older physiological stage on day 56 and day 84, we also evaluated the efficacy of long-term PTX administration on combating the senescence-associated with age growth (Figure 7). Sustained PTX treatment continued to inhibit BLM-induced cellular senescence while partially blocking the age-related changes in mice (Figure 7). Mice could benefit from long-term PTX administration because of the suppression of fibrosis and senescence development. Based on these research findings, we believed that the early and timely initiation and continuous therapy administration could bring more benefits to clinical patients with IPF when implementing anti-senescence-related treatment strategies.

Although we had confirmed the effectiveness of PTX treatment in animal experiments, to confirm that the therapeutic effect of PTX is also feasible in human background, we also conducted a series of *in vitro* experiments using human embryonic lung fibroblast HFL1 (Figure 8). Normal HFL1 cells and senescent HFL1 cells showed different responses to gradient concentrations of PTX treatment (Figure 8). Appropriate PTX concentration could effectively eliminate senescent HFL1 cells, and the gene expression related to fibrosis and senescence in aging HFL1 cells was also significantly downregulated during the PTX therapy (Figure 8). It could be said that these were quite optimistic *in vitro* experimental results. Meanwhile, when we considered the subsequent research transformation, *in vitro* experiments were just the beginning. We also needed to consider some realistic problems: previous clinical trials often reported that gastrointestinal reactions might accompany PTX treatment; the incidence rate of IPF disease was relatively low, and it was often difficult to get enough sample size in clinical research. More studies would be required before we know how to deal with these difficulties.

In our animal experiments *in vivo*, PTX treatment started the next day after BLM infusion showed the most significant advantage, indicating that it was advantageous to start treatment as soon as possible. In clinical practice, this might mean that intervention should be carried out once IPF is suspected or diagnosed as soon as possible. But it must be mentioned that intervention given before BLM-induced fibrosis formation possibly has a certain impact on the overall modeling results of BLM. However, when PTX treatment started on day 15, it also performed very strongly. The pity was that, although PTX administration effectively stopped the progression of pulmonary fibrosis, it seemed unable to reverse the existing fibrosis lesions or the destroyed alveolar septum in the lung tissue. And this was a crucial point that the current anti-fibrosis drugs for IPF were still unable to overcome. Our research was committed to achieving maximum advantage by starting treatment as early as possible. In fibrous lung diseases such as IPF, we might not be able to eliminate the fibrosis that has been formed. However, for fibrosis that has not yet formed, we were trying to find possible strategies to delay the arrival of a bad outcome.

On the other hand, the BLM model was not perfect itself. Previous studies have raised concerns about the shortcomings of BLM animal models of IPF (Schafer et al., 2017). The BLM-induced mouse pulmonary fibrosis model is currently recognized as the most widely used animal model of IPF. The fibrosis simulated by BLM in mice has the most typical characteristics of clinical patients with IPF. However, it had the defects of instability and self-limiting. Researchers have been working to find better, more stable animal models of IPF. However, as we have discussed before, the pathogenesis of IPF itself could not be directly generalized to some environmental factor or genetic abnormality; otherwise, it cannot be called “idiopathic” (Lin and Xu, 2020). Perhaps better animal models of IPF can be established by changing the type of modeling drugs or experimental animals.

**FIGURE 9:**
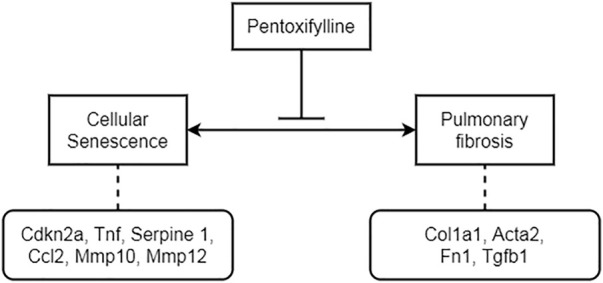
The potential mechanism of PTX inhibiting BLM-induced pulmonary fibrosis by regulating cellular senescence.

In general, PTX inhibited the formation of pulmonary fibrosis and regulated the occurrence of cellular senescence both in mice and in human fibroblasts (Figure 9). In mice, these therapeutic effects were closely related to the timely initiation and continuous administration of PTX therapy. Long-term PTX administration contributed to the remission of pulmonary fibrosis induced by BLM and countered the age-related senescence in lung tissue. These results illustrated the excellent performance of PTX in anti-fibrosis and anti-senescence works, suggesting the potential mechanisms that PTX inhibited pulmonary fibrosis by regulating cellular senescence.

## Data Availability

The datasets presented in this study can be found in online repositories. The names of the repository/repositories and accession number(s) can be found in the article/Supplementary Material.

## Supplementary Material

The Supplementary Material for this article can be found online at: https://www.frontiersin.org/articles/10.3389/fphar.2022.848263/full#supplementary-material

Supplementary Figure S1 The difference of mice body weight in each group on day 7, day 14, day 21, and day 28, compared with their body weight on day 0 (*n* = 4 for each group). Statistical significance is tested by one-way analysis of variance (ANOVA) with Tukey’s post-hoc test, **p* < 0.05, ***p* < 0.01, ****p* < 0.001, *****p* < 0.0001.

Supplementary Figure S2 Representative microscopic results of HE trichrome staining in the frozen sections of mice lung tissue in each group on day 14, day 21, and day 28 (*n* = 4 for each group). Original magnifications: ×200. Scale bars = 50 μm. Arrows indicate the pathological features of respective stains.

## References

[B1] Alam S. Nazmul Hasan S. Mustafa G. Alam M. Kamal M. Ahmad N. (2017). Effect of Pentoxifylline on Histological Activity and Fibrosis of Nonalcoholic Steatohepatitis Patients: A One Year Randomized Control Trial. J. Transl Int. Med. 5 (3), 155–163. 10.1515/jtim-2017-0021 29085788 PMC5655462

[B2] Álvarez D. Cárdenes N. Sellarés J. Bueno M. Corey C. Hanumanthu V. S. (2017). IPF Lung Fibroblasts Have a Senescent Phenotype. Am. J. Physiology-Lung Cell Mol. Physiol. 313 (6), L1164–L1173. 10.1152/ajplung.00220.2017 PMC6148001 28860144

[B3] Ashcroft T. Simpson J. M. Timbrell V. (1988). Simple Method of Estimating Severity of Pulmonary Fibrosis on a Numerical Scale. J. Clin. Pathol. 41 (4), 467–470. 10.1136/jcp.41.4.467 3366935 PMC1141479

[B4] Aygenc E. Celikkanat S. Kaymakci M. Aksaray F. Ozdem C. (2004). Prophylactic Effect of Pentoxifylline on Radiotherapy Complications: a Clinical Study. Otolaryngol. Head Neck Surg. 130 (3), 351–356. 10.1016/j.otohns.2003.08.015 15054378

[B5] Baker D. J. Wijshake T. Tchkonia T. LeBrasseur N. K. Childs B. G. van de Sluis B. (2011). Clearance of p16Ink4a-Positive Senescent Cells Delays Ageing-Associated Disorders. Nature 479 (7372), 232–236. 10.1038/nature10600 22048312 PMC3468323

[B6] Ballester B. Milara J. Cortijo J. (2020). Pirfenidone Anti-fibrotic Effects Are Partially Mediated by the Inhibition of MUC1 Bioactivation. Oncotarget 11 (15), 1306–1320. 10.18632/oncotarget.27526 32341751 PMC7170494

[B7] Baumgartner K. B. Samet J. M. Coultas D. B. Stidley C. A. Hunt W. C. Colby T. V. (2000). Occupational and Environmental Risk Factors for Idiopathic Pulmonary Fibrosis: a Multicenter Case-Control Study. Collaborating Centers. Am. J. Epidemiol. 152 (4), 307–315. 10.1093/aje/152.4.307 10968375

[B8] Bravo-Cuellar A. Ortiz-Lazareno P. C. Lerma-Diaz J. M. Dominguez-Rodriguez J. R. Jave-Suarez L. F. Aguilar-Lemarroy A. (2010). Sensitization of Cervix Cancer Cells to Adriamycin by Pentoxifylline Induces an Increase in Apoptosis and Decrease Senescence. Mol. Cancer 9, 114. 10.1186/1476-4598-9-114 20482878 PMC2890603

[B9] Cho S. J. Moon J. S. Lee C. M. Choi A. M. Stout-Delgado H. W. (2017). Glucose Transporter 1-Dependent Glycolysis Is Increased during Aging-Related Lung Fibrosis, and Phloretin Inhibits Lung Fibrosis. Am. J. Respir. Cel Mol Biol 56 (4), 521–531. 10.1165/rcmb.2016-0225OC PMC5449513 27997810

[B10] Choi W. I. Dauti S. Kim H. J. Park S. H. Park J. S. Lee C. W. (2018). Risk Factors for Interstitial Lung Disease: a 9-year Nationwide Population-Based Study. BMC Pulm. Med. 18 (1), 96. 10.1186/s12890-018-0660-2 29866093 PMC5987651

[B11] Cook M. Johnson N. Zegzula H. D. Schray M. Glissmeyer M. Sorenson L. (2016). Prophylactic Use of Pentoxifylline (Trental) and Vitamin E to Prevent Capsular Contracture after Implant Reconstruction in Patients Requiring Adjuvant Radiation. Am. J. Surg. 211 (5), 854–859. 10.1016/j.amjsurg.2016.01.006 27016313

[B12] de Souza R. B. Macedo A. R. Kuruma K. A. Macedo P. A. Borges C. T. (2009). Pentoxyphylline in Association with Vitamin E Reduces Cutaneous Fibrosis in Systemic Sclerosis. Clin. Rheumatol. 28 (10), 1207–1212. 10.1007/s10067-009-1202-3 19468787

[B13] de Toledo-Morrell L. Morrell F. Fleming S. Cohen M. M. (1984). Pentoxifylline Reverses Age-Related Deficits in Spatial Memory. Behav. Neural Biol. 42 (1), 1–8. 10.1016/s0163-1047(84)90364-9 6508689

[B14] Delanian S. Chatel C. Porcher R. Depondt J. Lefaix J. L. (2011). Complete Restoration of Refractory Mandibular Osteoradionecrosis by Prolonged Treatment with a Pentoxifylline-Tocopherol-Clodronate Combination (PENTOCLO): a Phase II Trial. Int. J. Radiat. Oncol. Biol. Phys. 80 (3), 832–839. 10.1016/j.ijrobp.2010.03.029 20638190

[B15] Entzian P. Gerlach C. Gerdes J. Schlaak M. Zabel P. (1997). Pentoxifylline Inhibits Experimental Bleomycin-Induced Fibrosing Alveolitis. Pneumologie 51 (4), 375–380. 9221384

[B16] Entzian P. Zähringer U. Schlaak M. Gerlach C. Galle J. Zabel P. (1998). Comparative Study on Effects of Pentoxifylline, Prednisolone and Colchicine in Experimental Alveolitis. Int. J. Immunopharmacol 20 (12), 723–735. 10.1016/s0192-0561(98)00056-3 9877283

[B17] Fell C. D. Martinez F. J. Liu L. X. Murray S. Han M. K. Kazerooni E. A. (2010). Clinical Predictors of a Diagnosis of Idiopathic Pulmonary Fibrosis. Am. J. Respir. Crit. Care Med. 181 (8), 832–837. 10.1164/rccm.200906-0959OC 20056903 PMC2854332

[B18] Futran N. D. Trotti A. Gwede C. (1997). Pentoxifylline in the Treatment of Radiation-Related Soft Tissue Injury: Preliminary Observations. Laryngoscope 107 (3), 391–395. 10.1097/00005537-199703000-00022 9121320

[B19] Gorgoulis V. Adams P. D. Alimonti A. Bennett D. C. Bischof O. Bishop C. (2019). Cellular Senescence: Defining a Path Forward. Cell 179 (4), 813–827. 10.1016/j.cell.2019.10.005 31675495

[B20] Gothard L. Cornes P. Brooker S. Earl J. Glees J. Hall E. (2005). Phase II Study of Vitamin E and Pentoxifylline in Patients with Late Side Effects of Pelvic Radiotherapy. Radiother. Oncol. 75 (3), 334–341. 10.1016/j.radonc.2005.02.002 16086914

[B21] Haddad P. Kalaghchi B. Amouzegar-Hashemi F. (2005). Pentoxifylline and Vitamin E Combination for Superficial Radiation-Induced Fibrosis: a Phase II Clinical Trial. Radiother. Oncol. 77 (3), 324–326. 10.1016/j.radonc.2005.09.014 16236376

[B22] Hernandez-Flores G. Ortiz-Lazareno P. C. Lerma-Diaz J. M. Dominguez-Rodriguez J. R. Jave-Suarez L. F. Aguilar-Lemarroy Adel C. (2011). Pentoxifylline Sensitizes Human Cervical Tumor Cells to Cisplatin-Induced Apoptosis by Suppressing NF-Kappa B and Decreased Cell Senescence. BMC Cancer 11, 483. 10.1186/1471-2407-11-483 22074157 PMC3229613

[B23] Hu R. Yuan B. X. Su L. Z. Wei X. Z. Zhao L. M. Kang J. (2007). Pentoxifylline Promotes Learning and Memory Function of Aging Rats and Mice with Induced Memory Impairment. Nan Fang Yi Ke Da Xue Xue Bao 27 (11), 1734–1737. 18024303

[B24] Hutchinson J. P. McKeever T. M. Fogarty A. W. Navaratnam V. Hubbard R. B. (2014). Increasing Global Mortality from Idiopathic Pulmonary Fibrosis in the Twenty-First century. Ann. Am. Thorac. Soc. 11 (8), 1176–1185. 10.1513/AnnalsATS.201404-145OC 25165873

[B25] Iwai K. Mori T. Yamada N. Yamaguchi M. Hosoda Y. (1994). Idiopathic Pulmonary Fibrosis. Epidemiologic Approaches to Occupational Exposure. Am. J. Respir. Crit. Care Med. 150 (3), 670–675. 10.1164/ajrccm.150.3.8087336 8087336

[B26] Jacobson G. Bhatia S. Smith B. J. Button A. M. Bodeker K. Buatti J. (2013). Randomized Trial of Pentoxifylline and Vitamin E vs Standard Follow-Up after Breast Irradiation to Prevent Breast Fibrosis, Evaluated by Tissue Compliance Meter. Int. J. Radiat. Oncol. Biol. Phys. 85 (3), 604–608. 10.1016/j.ijrobp.2012.06.042 22846413

[B27] Justice J. N. Nambiar A. M. Tchkonia T. LeBrasseur N. K. Pascual R. Hashmi S. K. (2019). Senolytics in Idiopathic Pulmonary Fibrosis: Results from a First-In-Human, Open-Label, Pilot Study. EBioMedicine 40, 554–563. 10.1016/j.ebiom.2018.12.052 30616998 PMC6412088

[B28] Kaapa P. Raj J. U. Ibe B. O. Anderson J. (1991). Effect of Pentoxifylline in Rabbit Pulmonary Circulation: Influence of Age and Vasomotor Tone. Am. J. Physiol. 261 (4 Pt 2), H975–H981. 10.1152/ajpheart.1991.261.4.H975 1928416

[B29] Kaya V. Yazkan R. Yıldırım M. Doğuç D. K. Süren D. Bozkurt K. K. (2014). The Relation of Radiation-Induced Pulmonary Fibrosis with Stress and the Efficiency of Antioxidant Treatment: an Experimental Study. Med. Sci. Monit. 20, 290–296. 10.12659/MSM.890334 24556959 PMC3937037

[B30] Kedarisetty C. K. Bhardwaj A. Kumar G. Rastogi A. Bihari C. Kumar M. (2021). Efficacy of Combining Pentoxiphylline and Vitamin E versus Vitamin E Alone in Non-alcoholic Steatohepatitis- A Randomized Pilot Study. Indian J. Gastroenterol. 40 (1), 41–49. 10.1007/s12664-020-01131-x 33772456

[B31] Kholakiya Y. Jose A. Rawat A. Nagori S. A. Jacob S. Roychoudhury A. (2020). Surgical Management of Oral Submucous Fibrosis with "Seagull-Nasolabial Flap" Combined with Short-Term Oral Pentoxifylline for Preventing Relapse. J. Stomatol Oral Maxillofac. Surg. 121 (5), 512–516. 10.1016/j.jormas.2019.12.015 31904528

[B32] Kwon H. C. Kim S. K. Chung W. K. Cho M. J. Kim J. S. Kim J. S. (2000). Effect of Pentoxifylline on Radiation Response of Non-small Cell Lung Cancer: a Phase III Randomized Multicenter Trial. Radiother. Oncol. 56 (2), 175–179. 10.1016/s0167-8140(00)00221-8 10927136

[B33] Lee J. G. Shim S. Kim M. J. Myung J. K. Jang W. S. Bae C. H. (2017). Pentoxifylline Regulates Plasminogen Activator Inhibitor-1 Expression and Protein Kinase A Phosphorylation in Radiation-Induced Lung Fibrosis. Biomed. Res. Int. 2017, 1279280. 10.1155/2017/1279280 28337441 PMC5350299

[B34] Lin Y. Xu Z. (2020). Fibroblast Senescence in Idiopathic Pulmonary Fibrosis. Front Cel Dev Biol 8, 593283. 10.3389/fcell.2020.593283 PMC7723977 33324646

[B35] Parnetti L. Ciuffetti G. Mercuri M. Lupattelli G. Senin U. (1986). The Role of Haemorheological Factors in the Ageing Brain: Long-Term Therapy with Pentoxifylline ('Trental' 400) in Elderly Patients with Initial Mental Deterioration. Pharmatherapeutica 4 (10), 617–627. 3602014

[B36] Premkumar K. V. Chaube S. K. (2016). Increased Level of Reactive Oxygen Species Persuades Postovulatory Aging-Mediated Spontaneous Egg Activation in Rat Eggs Cultured *In Vitro* . *In Vitro* Cel Dev Biol Anim 52 (5), 576–588. 10.1007/s11626-016-0007-3 26896066

[B37] Raghu G. Collard H. R. Egan J. J. Martinez F. J. Behr J. Brown K. K. (2011). An Official ATS/ERS/JRS/ALAT Statement: Idiopathic Pulmonary Fibrosis: Evidence-Based Guidelines for Diagnosis and Management. Am. J. Respir. Crit. Care Med. 183 (6), 788–824. 10.1164/rccm.2009-040GL 21471066 PMC5450933

[B38] Raghu G. Weycker D. Edelsberg J. Bradford W. Z. Oster G. (2006). Incidence and Prevalence of Idiopathic Pulmonary Fibrosis. Am. J. Respir. Crit. Care Med. 174 (7), 810–816. 10.1164/rccm.200602-163OC 16809633

[B39] Raj J. U. Kaapa P. Anderson J. (1992). Age-related Differences in Vascular Effects of Pentoxifylline in Isolated Perfused Ferret Lungs. Dev. Pharmacol. Ther. 18 (1-2), 1–8. 10.1159/000480591 1483354

[B40] Rangarajan S. Bone N. B. Zmijewska A. A. Jiang S. Park D. W. Bernard K. (2018). Metformin Reverses Established Lung Fibrosis in a Bleomycin Model. Nat. Med. 24 (8), 1121–1127. 10.1038/s41591-018-0087-6 29967351 PMC6081262

[B41] Rangarajan S. Kurundkar A. Kurundkar D. Bernard K. Sanders Y. Y. Ding Q. (2016). Novel Mechanisms for the Antifibrotic Action of Nintedanib. Am. J. Respir. Cel Mol Biol 54 (1), 51–59. 10.1165/rcmb.2014-0445OC PMC4742925 26072676

[B42] Roman J. Ritzenthaler J. D. Bechara R. Brown L. A. Guidot D. (2005). Ethanol Stimulates the Expression of Fibronectin in Lung Fibroblasts via Kinase-dependent Signals that Activate CREB. Am. J. Physiol. Lung Cel Mol Physiol 288 (5), L975–L987. 10.1152/ajplung.00003.2004 15653713

[B43] Sadaksharam J. Mahalingam S. (2017). Evaluation of Oral Pentoxifylline in the Management of Oral Submucous Fibrosis - an Ultrasonographic Study. Contemp. Clin. Dent 8 (2), 200–204. 10.4103/ccd.ccd_1192_16 28839403 PMC5551322

[B44] Schafer M. J. White T. A. Iijima K. Haak A. J. Ligresti G. Atkinson E. J. (2017). Cellular Senescence Mediates Fibrotic Pulmonary Disease. Nat. Commun. 8, 14532. 10.1038/ncomms14532 28230051 PMC5331226

[B45] Seidler N. W. Swislocki N. I. (1992). The Effects of Pentoxifylline on the Plasma Membrane Ca2+ ATPase in Age-Separated Rat and Human Erythrocytes. J. Clin. Pharmacol. 32 (4), 332–337. 10.1002/j.1552-4604.1992.tb03844.x 1533232

[B46] Seluanov A. Vaidya A. Gorbunova V. (2010). Establishing Primary Adult Fibroblast Cultures from Rodents. J. Vis. Exp. 44, 2033. 10.3791/2033 PMC3185624 20972406

[B47] Serag-Eldin Y. M. A. Mahmoud W. H. Gamea M. M. Hegab D. S. (2021). Intralesional Pentoxifylline, Triamcinolone Acetonide, and Their Combination for Treatment of Keloid Scars. J. Cosmet. Dermatol. 20 (10), 3330–3340. 10.1111/jocd.14305 34138506

[B48] Solerte S. B. Ceresini G. Ferrari E. Fioravanti M. (2000). Hemorheological Changes and Overproduction of Cytokines from Immune Cells in Mild to Moderate Dementia of the Alzheimer's Type: Adverse Effects on Cerebromicrovascular System. Neurobiol. Aging 21 (2), 271–281. 10.1016/s0197-4580(00)00105-6 10867211

[B49] Tan A. Martinez Luna O. Glass D. A. 2nd (2020). Pentoxifylline for the Prevention of Postsurgical Keloid Recurrence. Dermatol. Surg. 46 (10), 1353–1356. 10.1097/DSS.0000000000002090 31397783 PMC7164298

[B50] Wang Y. Kang Y. Qi C. Zhang T. Zhao H. Ji X. (2020). Pentoxifylline Enhances Antioxidative Capability and Promotes Mitochondrial Biogenesis for Improving Age-Related Behavioral Deficits. Aging (Albany NY) 12 (24), 25487–25504. 10.18632/aging.104155 33231568 PMC7803534

[B51] Ward W. F. Molteni A. Ts'ao C. H. Kim Y. T. Hinz J. M. (1992). Radiation Pneumotoxicity in Rats: Modification by Inhibitors of Angiotensin Converting Enzyme. Int. J. Radiat. Oncol. Biol. Phys. 22 (3), 623–625. 10.1016/0360-3016(92)90890-t 1735701

[B52] Yosef R. Pilpel N. Tokarsky-Amiel R. Biran A. Ovadya Y. Cohen S. (2016). Directed Elimination of Senescent Cells by Inhibition of BCL-W and BCL-XL. Nat. Commun. 7, 11190. 10.1038/ncomms11190 27048913 PMC4823827

[B53] Yousefzadeh M. J. Zhu Y. McGowan S. J. Angelini L. Fuhrmann-Stroissnigg H. Xu M. (2018). Fisetin Is a Senotherapeutic that Extends Health and Lifespan. EBioMedicine 36, 18–28. 10.1016/j.ebiom.2018.09.015 30279143 PMC6197652

