# Supplementary figures and images for: Pentoxifylline Inhibits Pulmonary Fibrosis by Regulating Cellular Senescence in Mice
Source: Front Pharmacol. 2022 May 19;13:848263. doi: 10.3389/fphar.2022.848263 (PMC9160723; doi:10.3389/fphar.2022.848263)

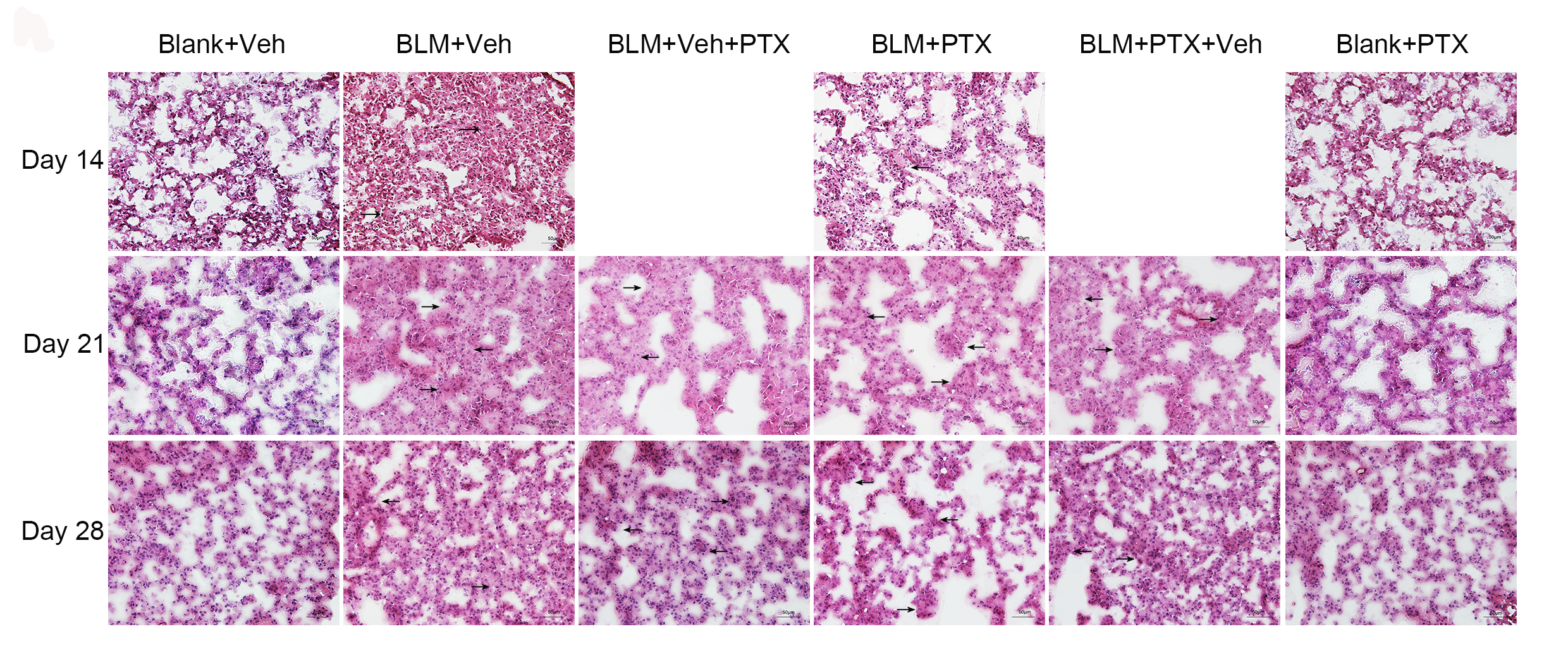

Supplement: Supplementary file 1 [file Image2.TIF]

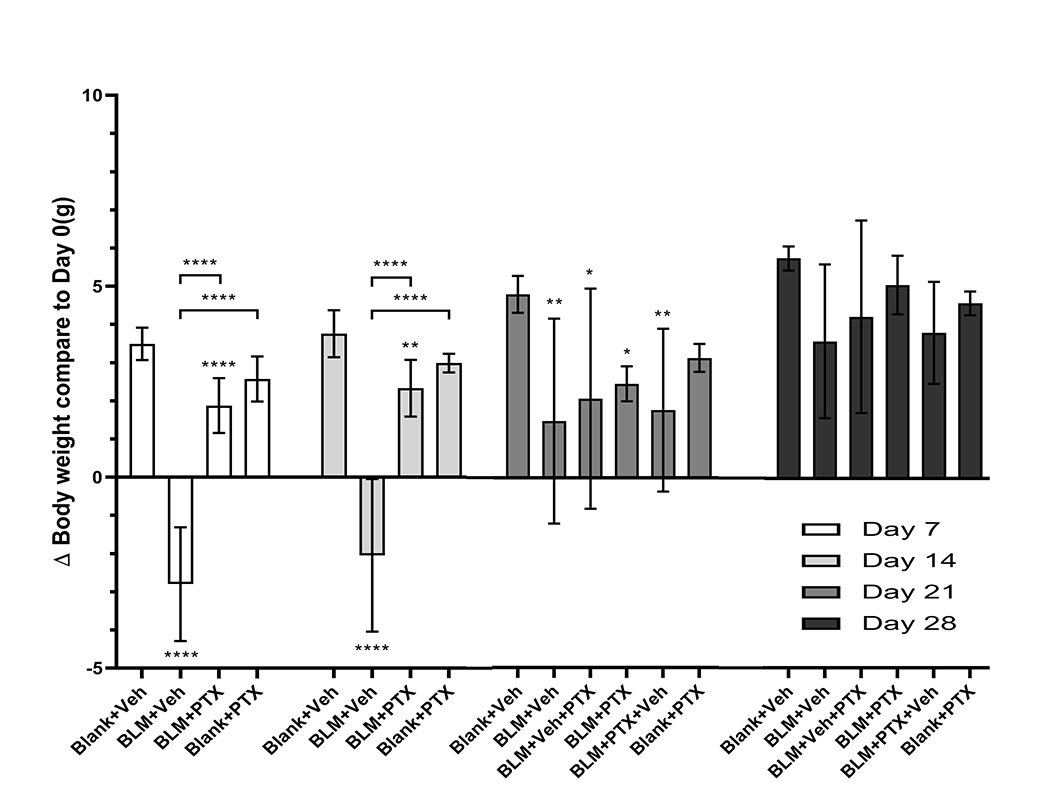

Supplement: Supplementary file 2 [file Image1.TIF]
